# Killer to cure: Expression and production costs calculation of tobacco plant‐made cancer‐immune checkpoint inhibitors

**DOI:** 10.1111/pbi.14034

**Published:** 2023-03-18

**Authors:** Laura A. Ridgley, Nicole Falci Finardi, Benjamin B. Gengenbach, Patrick Opdensteinen, Zack Croxford, Julian K.‐C. Ma, Mark Bodman‐Smith, Johannes F. Buyel, Audrey Y.‐H. Teh

**Affiliations:** ^1^ Institute for Infection and Immunity, St. George's University of London London UK; ^2^ Institute for Cancer Vaccines and Immunotherapy London UK; ^3^ Fraunhofer Institute for Molecular Biology and Applied Ecology IME Aachen Germany; ^4^ Institute for Molecular Biotechnology RWTH Aachen University Aachen Germany; ^5^ Department of Biotechnology (DBT), Institute of Bioprocess Science and Engineering (IBSE) University of Natural Resources and Life Sciences, Vienna (BOKU) Vienna Austria

**Keywords:** Immune checkpoint inhibitors, Cancer, Plant expression vector, production cost model, T‐cell, NK cell

## Abstract

Immune checkpoint inhibitors (ICIs) have achieved huge clinical success. However, many still have limited response rates, and are prohibitively costly. There is a need for effective and affordable ICIs, as well as local manufacturing capacity to improve accessibility, especially to low‐to‐middle income countries (LMICs). Here, we have successfully expressed three key ICIs (anti‐PD‐1 Nivolumab, anti‐NKG2A Monalizumab, and anti‐LAG‐3 Relatimab) transiently in *Nicotiana benthamiana* and *Nicotiana tabacum* plants. The ICIs were expressed with a combination of different Fc regions and glycosylation profiles. They were characterized in terms of protein accumulation levels, target cell binding, binding to human neonatal Fc receptors (hFcRn), human complement component C1q (hC1q) and various Fcγ receptors, as well as protein recovery during purification at 100 mg‐ and kg‐scale. It was found that all ICIs bound to the expected target cells. Furthermore, the recovery during purification, as well as Fcγ receptor binding, can be altered depending on the Fc region used and the glycosylation profiles. This opens the possibility of using these two parameters to fine‐tune the ICIs for desired effector functions. A scenario‐based production cost model was also generated based on two production scenarios in hypothetical high‐ and low‐income countries. We have shown that the product accumulation and recovery of plant production platforms were as competitive as mammalian cell‐based platforms. This highlights the potential of plants to deliver ICIs that are more affordable and accessible to a widespread market, including LMICs.

## Introduction

Cancer is a leading cause of death worldwide, with its incidence and mortality only set to increase (Sung *et al*., 2021). A complex interplay occurs between the immune system and tumour cells—the balance between these opposing sides plays a crucial role in determining the outcome. Tumour cells can often be recognized *via* tumour antigens and subsequently eliminated by the immune system. However, due to their high rate of cell division and mutational burden, this selective pressure can result in new variants of tumour cells which are able to escape elimination (Dunn *et al*., 2004; Hanahan and Weinberg, 2011; Mittal *et al*., 2014).

Mechanisms employed by tumour cells to evade immune cells include: (1) the production of an immunosuppressive tumour microenvironment (TME; Labani‐Motlagh *et al*., 2020); (2) the avoidance of recognition by downregulating tumour antigens, Major histocompatibility complex (MHC) molecules and/or costimulatory molecules (Hicklin *et al*., 1999; Restifo *et al*., 1993); and (3) the expression of inhibitory molecules for immune cell suppression (Dong *et al*., 2002). The interaction of these inhibitory ligands with immune checkpoints regulates the timing and intensity of immune responses. ICIs interfere with this negative regulatory pathway and restore anti‐tumour functions with considerable clinical success in numerous cancers including non‐small cell lung cancer (NSCLC) and melanoma (Garon *et al*., 2015; Hodi *et al*., 2010; Topalian *et al*., 2012).

One of the most successful ICIs targets the programmed cell death 1 (PD‐1) molecule, a key checkpoint on T‐cells which dampens responses (including proliferation, cytokine production and cytotoxicity) when bound to programmed death ligand 1 (PD‐L1) or PD‐L2 on tumour cells (Patsoukis *et al*., 2020). Many tumours express high levels of PD‐L1, suggested to correspond to disease severity and outcome (Hou *et al*., 2014; Mu *et al*., 2011; Qing *et al*., 2015; Shi *et al*., 2013). Nivolumab, a monoclonal antibody against PD‐1 has shown considerable success in treatment and has been licensed for use in several cancers (Gettinger *et al*., 2015; Tang *et al*., 2018; Weber *et al*., 2015).

Other checkpoints on T‐cells include cytotoxic T‐lymphocyte‐associated antigen 4 (CTLA‐4) and CD28, which share the CD86 and CD80 ligands on Antigen Presenting Cells (Rowshanravan *et al*., 2018). Anti‐CTLA‐4/anti‐PD‐1 combination therapies have been approved for the treatment of different types of cancers (Rotte, 2019; Seidel *et al*., 2018). Lymphocyte activation gene‐3 (LAG‐3) binds MHC class II, and ICIs against LAG‐3 are currently being investigated alone or in combination with existing ICIs in numerous clinical trials (Feeney *et al*., 2019; Lakhani *et al*., 2020; Lipson *et al*., 2018). An emerging checkpoint NKG2A, primarily expressed on natural killer (NK) cells but also tumour infiltrating lymphocytes (TIL), has been shown to bind human leukocyte antigen E (HLA‐E) which is frequently upregulated on tumour cells (André *et al*., 2018; Io Monaco *et al*., 2011; Kamiya *et al*., 2019). Co‐expression of NKG2A and PD‐1 has been documented on CD8+ TILs, and current investigations are exploring the anti‐tumour potential of this combination of ICIs in clinical trials (André *et al*., 2018; Montfoort *et al*., 2018).

Despite the huge clinical success of ICIs, response rates only reach a maximum of 40%, and many responsive patients can go on to acquire resistance (Borghaei *et al*., 2015; Garon *et al*., 2015; Larkin *et al*., 2015). For this reason, a combination of ICIs may be beneficial in increasing patient response rates (Khair *et al*., 2019; Ma *et al*., 2021). A major barrier to this is the price of therapy, with a course per patient costing ~£70 000 (Centers for Medicare and Medicaid Services (CMS), 2021; Doyle, 2016; National Institute for Health Care and Excellence, 2021; Verma *et al*., 2018). Unlimited use of these antibodies is prohibitively costly, especially in LMICs which are already ill‐equipped to deal with the current disproportionate disease burden and cancer‐related mortality (American Cancer Society, 2021; Kanavos, 2006; Sung *et al*., 2021; Torre *et al*., 2016).

Production of pharmaceuticals using the plant manufacturing platform is gaining momentum worldwide, with ELELYSO® (a recombinant glucocerebrosidase made in carrot cells) for the treatment of Gaucher's disease in clinical use (Zimran *et al*., 2018) and COVIFENZ® [a SARS‐CoV2 virus‐like‐particle‐based vaccine made in tobacco plants (Ward *et al*., 2021)] approved for emergency use. There are many more in clinical trials, including an influenza vaccine (Pillet *et al*., 2016), and the anti‐Ebola cocktail ZMapp® (Davey *et al*., 2016).

The plant manufacturing system has a number of advantages over mammalian cell platforms including lower initial investment, lower cultivation costs, easily scalable production, and easily transferable technology suitable for use in LMICs (Paul *et al*., 2013). Plant‐made recombinant products are also free from contaminating human pathogens, animal‐derived products and endotoxins (Paul *et al*., 2013). Furthermore, proteins produced in plants possess a more homogenous glycosylation profile compared to mammalian‐cell‐made ones, and efforts have been made to humanize the glycosylation pathway of a variety of plant species (Göritzer *et al*., 2022; Jansing *et al*., 2019; Kallolimath *et al*., 2016; Mercx *et al*., 2017; Montero‐Morales and Steinkellner, 2018; Schneider *et al*., 2015). These factors will be particularly important for attracting investment, especially from LMICs.

The aim of this study is to explore the feasibility of producing ICIs in a plant manufacturing system. More specifically, we aim to determine the protein accumulation and recovery, ligand binding characteristics, functional abilities, and manufacturing cost of these plant‐derived ICIs. We will also demonstrate the use of an IgG1‐based constant region to increase purification recovery and reduce Fcγ receptor binding. This will provide the first proof of concept that aims to deliver affordable ICIs to a more widespread market using the plant manufacturing system.

## Results

### Characterisation and purification recovery of ICIs

ICIs Monalizumab (anti‐NKG2A), Nivolumab (anti‐PD‐1), and Relatimab (anti‐LAG‐3) were successfully expressed transiently in *N. benthamiana* and *N. tabacum* using the plant expression system MIDAS‐P (Pinneh *et al*., 2022; Teh *et al*., 2021). These ICIs were initially expressed in the IgG4k format with S228P mutation (IgG4P; Silva *et al*., 2015) to reduce half antibody formation and Fab‐arm exchange. This format was consistent with those in clinical use or in clinical trials which were made in mammalian cells (Ascierto *et al*., 2017; Topalian *et al*., 2012; Van Hall *et al*., 2019). For reasons mentioned in the next section, the ICIs were also expressed in the IgG1 format with L324A/L325A/P329G (LALAPG) mutation (IgG1AAG; Schlothauer *et al*., 2016) to improve purification recovery and reduce Fcγ receptor binding.

Non‐reducing PAGE gel (Figure S1A) and non‐reducing anti‐gamma Western blot (Figure S2A) indicated that the plant‐made ICIs had assembled correctly, were of the correct size, and had similar separation profiles to their mammalian‐cell‐made counterparts. In the reducing PAGE gel, two bands representing the heavy and light chains were seen (Figure S1B). Their identities were confirmed by reducing Western blots using anti‐human gamma (Figure S2A, black arrow and S2B, grey arrow) and anti‐human kappa antisera (Figure S2B, white arrow). Furthermore, Western blots using anti‐β1,2‐xylose (Figure S3A) and anti‐α1,3‐fucose (Figure S3B) antisera indicated the absence of both these sugars on ICIs produced in glycomodified plants. In contrast, ICIs produced in plants with wild‐type (WT) glycosylation retained both these sugars.

To quantify protein accumulation level, the ICIs were infiltrated on its own or with a separate construct containing the Tomato Bushy Stunt Virus (TBSV) P19 post‐transcriptional gene silencing suppressor, which has previously shown to increase accumulation levels (Voinnet *et al*., 2003). ICIs extracted from infiltrated leaves were quantified using Enzyme‐linked immunosorbent assay (ELISA). When expressed on their own, Nivolumab had the highest accumulation level at 0.073 g/kg leaf fresh weight (LFW), followed by Relatimab and Monalizumab (Table 1). Furthermore, there were no significant differences between the accumulation levels for the IgG4P and IgG1AAG versions of Nivolumab (Table 1). Co‐expression with P19 helped to improve accumulation levels of all constructs, with the levels around 5–10 times higher compared to those expressed without P19 (Table 1). For example, the accumulation level of Nivolumab increased to 0.77 g/kg with P19 co‐expression.

**Table 1:** Protein accumulation levels of Immune checkpoint inhibitors (ICIs) Nivolumab, Monalizumab and Relatimab with an IgG4 S228P (IgG4P) Fc scaffold, as well as Nivolumab variable regions with an IgG1 L324A/L325A/P329G (Nivolumab IgG1AAG) scaffold*.* ICIs were expressed transiently with or without TBSV P19 silencing suppressor in *N. benthamiana* with WT glycosylation. Leaves were harvested at 6 dpi. Protein accumulation levels represent the mean, minimum and maximum of six biological repeats

ICI	Target	Protein accumulation level (g/kg LFW)	
		Without P19	With P19
Nivo‐IgG4P	PD‐1	0.073 ± 0.007	0.770 ± 0.192
Nivo‐IgG1AAG	PD‐1	0.06 ± 0.01	0.557 ± 0.128
Monalizumab	NKG2A	0.02 ± 0.004	0.173 ± 0.090
Relatimab	LAG‐3	0.043 ± 0.005	0.416 ± 0.036

### Improving purification recovery of Nivolumab using IgG1AAG constant region

Nivolumab was selected based on their accumulation level (Table 1) for further characterization of protein recovery during purification. At 100 mg‐scale, purification runs of Nivolumab with IgG4P constant region showed more than 90% loss after Protein A affinity chromatography, with most of the protein present in the flowthrough fraction (Table 2A). Subsequent analysis using surface plasmon resonance (SPR) indicated that the S228P mutation reduced the affinity of both mammalian cell‐ and plant‐made ICIs to Protein A (Figure S4B,C), compared to non‐mutated IgG4 (Figure S4A). It seemed that the S228P mutation, which was introduced to prevent Fab‐arm exchange in IgG4 antibodies (Silva *et al*., 2015), had caused faster dissociation from Protein A. This most probably contributed to the drop in recovery when using Protein A purification. The binding kinetics of IgG4P ICIs to Protein G and L were also investigated, but they did not show any improvement in binding (data not shown).

**Table 2:** Protein recovery at different stages of Protein A purification at (A) 100 mg‐scale and (B) at kg‐scale. Two Nivolumab formats were used: the IgG4k S228P (IgG4P) and the IgG1 L324A/L325A/P329G (IgG1AAG). All plant‐made ICIs were co‐expressed with TBSV P19 post‐transcriptional gene silencing suppressor. In the 100 mg‐scale experiment, ELISA was used to measure protein concentration using anti‐kappa antiserum (The Binding Site, UK) for capture, and horseradish peroxidase‐conjugated anti‐IgG1 Fc (The Binding Site, UK) or anti‐IgG4 (Sigma) antiserum for detection. IgG1k or IgG4k (Sigma) was used as the standard. In the kg‐scale experiment, SPR with a Protein A coated CM5 chip and IgG1k standard was used.

Process step	Protein accumulation or recovery (g/kg LFW)		Overall recovery	
	IgG1AAG	IgG4P	IgG1AAG	IgG4P
(A)				
Crude extract	0.43 ± 0.019	0.678 ± 0.004	1.00	1.00
Clarified extract	0.417 ± 0.037	0.599 ± 0.059	0.97	0.80
Post‐filtration	0.410 ± 0.03	0.604 ± 0.052	0.95	0.89
Eluted	0.39 ± 0.019	0.014 ± 0.007	0.91	0.02
Flowthrough	n.d.	0.414 ± 0.044	–	–
Post‐centricon	0.359 ± 0.009	0.062 ± 0.02	0.83	0.09

Process step	Purity (g/g TSP)		Protein accumulation or recovery (g/kg LFW)		Overall recovery		Step recovery	
	IgG1AAG	IgG4P	IgG1AAG	IgG4P	IgG1AAG	IgG4P	IgG1AAG	IgG4P
(B)								
Homogenate	0.05	0.00	0.466	n.a.	1.00	n.a.	n.a.	n.a.
Bag filtrate	0.06	0.01	0.245	0.228	0.39	1.00	0.39	n.a.
Depth filtrate	0.07	0.01	0.126	0.171	0.20	0.75	0.52	0.75
Sterile filtrate	0.05	0.03	0.109	0.151	0.27	0.66	0.88	0.88
Elution	>0.95	>0.95	0.163	0.110	0.36	0.49	>0.95	0.73

The variable regions of all ICIs were subsequently cloned into an IgG1 constant region with L324A/L325A/P329G mutation (IgG1AAG). This mutation has been shown to reduce the binding of IgG1 antibodies to Fc receptors (Schlothauer *et al*., 2016). PAGE gel and Western blot showed successful expression and assembly of plant‐made IgG1AAG Nivolumab (Figures S1 and S2). SPR analysis also indicated that ICIs with an IgG1AAG constant region dissociated more slowly from Protein A (Figure S4E) compared to both mammalian‐cell and plant‐made IgG4P ICIs (Figure S4B,C). There were no differences in the binding kinetics of Protein A to IgG1AAG ICIs (Figure S4E) compared to non‐modified IgG1 (Figure S4D).

We then confirmed the performance of Nivolumab with IgG4P and IgG1AAG constant regions in a scalable extraction and purification setting as previously described (Buyel and Fischer, 2014). The main differences between the extraction and purification processes at 100‐mg and kg scale were (1) the extraction method (blender vs. blade‐based homogenizer); and (2) the clarification method (centrifugation/filtration vs. bag/depth/sterile filtration). Interestingly, using the IgG1AAG constant region did indeed increase the step recovery compared to IgG4P (Table 2B) in the kg‐scale setting. However, the overall product recovery decreased by about 10% because more IgG1AAG was lost during the clarification stage, *e.g*. depth filtration.

### Plant‐derived ICIs were able to bind to complementary ligands on immune cells

Following the successful production and characterisation of plant‐derived immune checkpoint inhibitors, the next aim was to assess the binding to their respective ligands on immune cell subsets. Binding of Nivolumab and Relatimab to activated CD3+ T‐cells was assessed, while binding of Monalizumab was assessed on activated NK cells.

**Figure 1:**
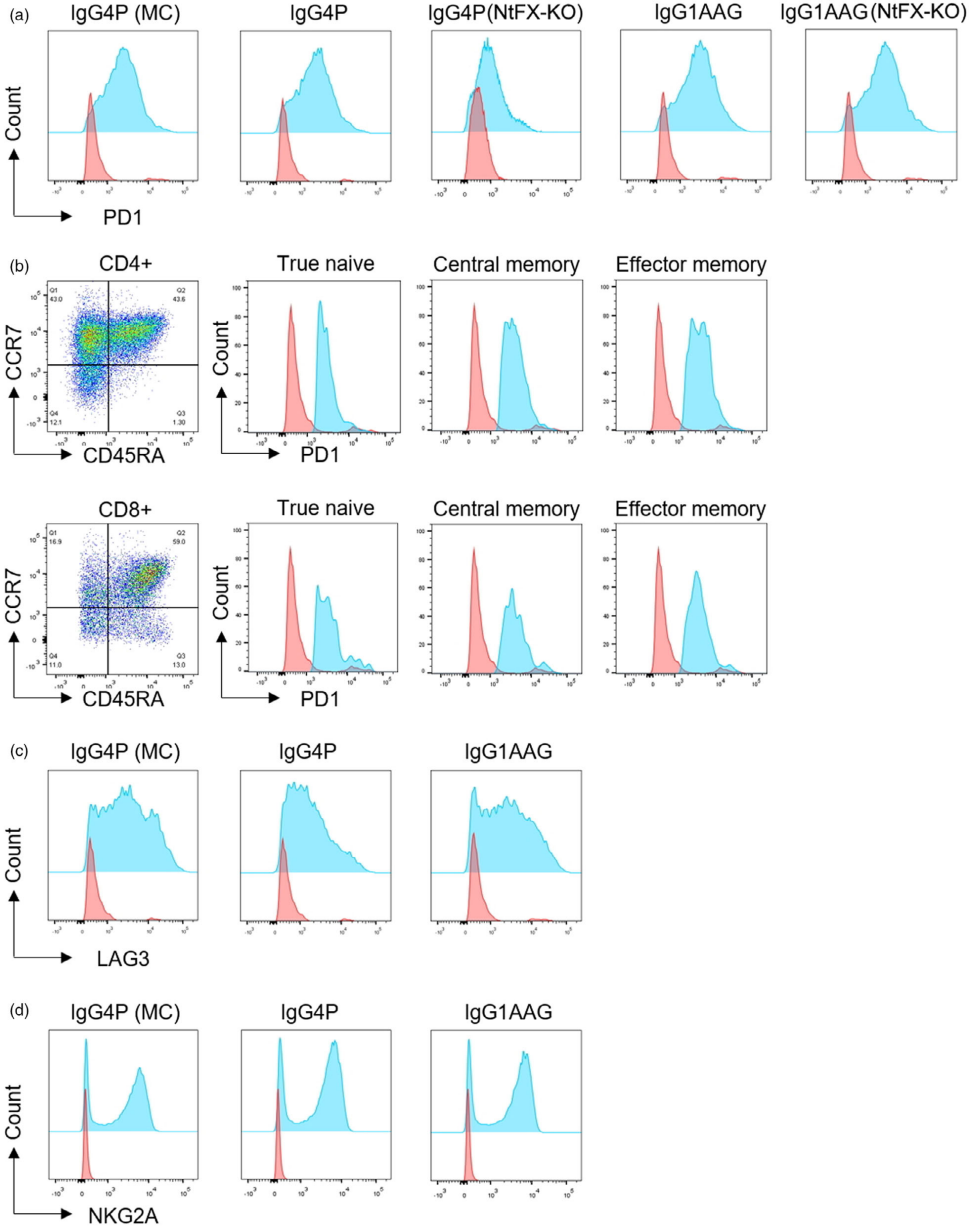
Specificity of plant‐produced ICIs compared to controls. Binding of plant‐made IgG4P and IgG1AAG ICIs to human CD3+ T‐cells activated by anti‐CD2/CD3/CD28 beads, or CD56+ NK cells activated with IL‐15 and IL‐2, both for 3 days. (a) Binding of mammalian‐cell‐made IgG4P Nivolumab (MC); plant‐made, WT glycosylated, IgG4P Nivolumab (IgG4P); plant‐made afucosylated and axylosylated IgG4P Nivolumab (IgG4P NtFX‐KO); plant‐made, WT glycosylated, IgG1AAG Nivolumab (IgG1AAG); and plant‐made Nivolumab IgG1AAG made in glycomodified plants (IgG1AAG NtFX‐KO) (blue) was detected by a secondary antibody against IgG, and assessed compared to binding of IgGk isotype control (red). (b) Binding of plant‐made Nivolumab was examined on subsets of CD4+ and CD8+ T‐cells including Naïve T‐cells (TN; CD45RA + CCR7+), Central Memory T‐cells (CM; CD45RA−CCR7+) and Effector Memory T‐cells (EM; CD45RA−CCR7−). (c) Binding of mammalian‐cell‐made IgG4P Relatimab (MC); plant‐made, WT glycosylated, IgG4P Relatimab (IgG4P); and IgG1AAG Relatimab (IgG1AAG) (blue) was detected by a secondary antibody against IgG and assessed compared to binding of IgGk isotype control (red). (d) Binding of mammalian‐cell‐made IgG4P Monalizumab (MC); plant‐made, WT glycosylated, IgG4P Monalizumab (IgG4P); and IgG1AAG Monalizumab (IgG1AAG) (blue) was detected by a secondary antibody against IgG compared to binding of hIgG4k or hIgG1k isotype control (red). Representative plots from six donors are shown.

Flow cytometric analysis revealed binding of both IgG4P and IgG1AAG Nivolumab to activated CD3+ T‐cells compared to IgG controls (Figure 1a). We have tested two versions each of IgG4P and IgG1AAG Nivolumab – one with WT plant glycosylation; another with axylosylated and afucosylated glycosylation which are produced in NtFX‐KO plant lines. NtFX‐KO is an *N. tabacum* cv. SR1 plant line with their α1,3‐Fucosyltransferase and β1,2‐Xylosyltransferase genes knocked out using CRISPR/Cas9 (Göritzer *et al*., 2022). Glycosylation and the Fc region of plant‐made ICIs did not affect ligand binding, as similar levels of binding were seen with IgG4P and IgG1AAG Nivolumab containing either WT or modified glycosylation (NtFX‐KO; Figure 1a). Binding of plant‐made Nivolumab was seen in both CD4+ and CD8+ T‐cells, with higher levels of binding seen on central memory (CM) and effector memory (EM) subsets, and less binding to the naïve (TN) cell subsets of either cell type (Figure 1b).

Analysis of Relatimab revealed binding of both IgG4P and IgG1AAG ICIs compared to IgG controls (Figure 1c). Furthermore, both IgG4P and IgG1AAG Monalizumab showed successful binding to activated CD56+ NK cells (Figure 1d).

### Binding of plant‐made ICIs to hFcRn

Surface plasmon resonance was used to determine the binding of IgG4P and IgG1AAG ICIs made in plants (with WT and modified glycosylation) to hFcRn. This was then compared to their mammalian cell‐made IgG4, IgG1, IgG4P or IgG1AAG counterparts.

CM5 chip coated with anti‐Fab binders was used to capture the ICIs. Different concentrations of hFcRn were flowed over the ICIs at pH 6.0 and pH 7.4 to determine the association and dissociation curve. Increased interaction of hFcRn with the Fc region at pH 6 is linked to improved half‐life, as hFcRn salvages IgGs from being degraded in the lysosome and releases them back into circulation (Petkova *et al*., 2006). All ICIs bound to hFcRn at *K*_*D*_ of 10^−7^–10^−8^ M at pH 6.0, which is the pH of the sorting endosomes where the IgGs bind to hFcRn (Tables 3 and S2A). There was no binding at pH 7.4, the physiological pH of the bloodstream where the IgGs are released by hFcRn (Tables 3 and S2B).

**Table 3:** Binding of ICIs to human hFcRn or Fcγ receptors. (A) Equilibrium dissociation constants (*K*_*D*_) of IgG4P and IgG1AAG ICIs from mammalian cells, glycomodified (NtFX‐KO) and wild‐type (WT) plants to hFcRn. IgG1k and IgG4k from human myeloma plasma (Sigma) were used as controls. Two biological replicates were performed for each experiment. (B) Binding response (expressed as Response Units or RUs) of IgG4P and IgG1AAG ICIs to human Fcγ receptors compared to IgG1k and IgG4k. Both flow cells of a CM5 chip were coated with anti‐His‐tag antibodies to ~600 RU. 5 μg/mL of His‐tagged receptors (R&D systems, NE Minneapolis, Minnesota) were captured on one flow cell, and 100 μg/mL of ICIs were flowed over both flow cells. Running buffer over captured receptors blanks was run before and after each experiment and the average was subtracted from the binding response. Three biological replicates were performed for each experiment. (C) *K*_*D*_ of IgG1AAG ICIs from mammalian cells, NtFX‐KO and WT plants to human FcγRI, RIIa, RIIb/c and RIIIa receptors. CM5 chips were coated with ~2000 RU of *S. aureus* Protein A (Sigma) on both flow cells. Experimental conditions were carried out as detailed in Table S1. IgG1k from human myeloma plasma and NtFX‐KO plant‐made human IgG1k were used as controls. Two biological replicates were performed for each experiment.

ICIs/antibodies	*K*_*D*_ (nM)	
	hFcRn (pH 6.0)	hFcRn (pH 7.4)
(A)		
IgG1 MC	143 ± 158	No binding
IgG1 NtFX‐KO	36 ± 20	No binding
IgG4 MC	90 ± 26	No binding
IgG4P MC	53 ± 52	No binding
IgG4P NtFX‐KO	60 ± 66	No binding
IgG4P WT	63 ± 73	No binding
IgG1AAG MC	99 ± 24	No binding
IgG1AAG NtFX‐KO	156 ± 61	No binding
IgG1AAG WT	85 ± 10	No binding

	ICIs/antibodies	Response units (RUs)			Mean ± SD
		Run 1	Run 2	Run 3	
(B)					
hFcγRI	IgG1 MC	155	145	183	161 ± 20
	IgG4 MC	147	185	164	165 ± 19
	IgG4P MC	175	125	159	153 ± 25
	IgG4P NtFX‐KO	124	104	170	133 ± 34
	IgG4P WT	142	176	123	147 ± 27
	IgG1AAG NtFX‐KO	1	0	2	1 ± 1
	IgG1AAG WT	0	0	1	0 ± 1
hFcγRIIa	IgG1 MC	142	144	115	133 ± 16
	IgG4 MC	22	45	13	27 ± 16
	IgG4P MC	92	80	36	70 ± 29
	IgG4P NtFX‐KO	70	86	75	77 ± 8
	IgG4P WT	19	14	12	15 ± 4
	IgG1AAG NtFX‐KO	0	0	0	0
	IgG1AAG WT	0	0	1	0 ± 1
hFcγRIIb/c	IgG1 MC	81	71	60	71 ± 10
	IgG4 MC	44	65	38	49 ± 14
	IgG4P MC	58	51	48	52 ± 5
	IgG4P NtFX‐KO	51	48	53	51 ± 2
	IgG4P WT	34	5	0	13 ± 18
	IgG1AAG NtFX‐KO	0	0	3	1 ± 2
	IgG1AAG WT	0	0	3	1 ± 2
hFcγRIIIa	IgG1 MC	570	574	480	541 ± 53
	IgG4 MC	15	26	0	13 ± 13
	IgG4P MC	57	74	22	51 ± 26
	IgG4P NtFX‐KO	213	341	223	259 ± 71
	IgG4P WT	57	23	10	30 ± 24
	IgG1AAG NtFX‐KO	34	11	66	37 ± 28
	IgG1AAG WT	0	0	0	0

ICIs/antibodies	Receptors			
	hFcγRI	hFcγRIIa	hFcγRIIb/c	hFcγRIIIa
(C)				
IgG1 MC	0.646 ± 0.811	309 ± 158	3358 ± 569	417 ± 245
IgG1 NtFX‐KO	0.152 ± 0.016	321 ± 436	3863 ± 3031	31 ± 13
IgG1AAG MC	2.82 ± 3.71	No binding	No binding	5250 ± 1993
IgG1AAG NtFX‐KO	5.48 ± 1.03	No binding	No binding	655 ± 899
IgG1AAG WT	138 ± 187	No binding	15 765 ± 9525	12 257 ± 16 325
	*K*_*D*_ (nM)			

### Binding of plant‐made ICIs to human Fcγ receptors

The IgG4P ICIs did not bind well to Protein A (Figure S4B,C). Therefore, we have used an SPR format which involved binding to His‐tagged receptors captured on a CM5 chip coated with anti‐Polyhistidine antibodies to compare the binding of both the IgG4P and IgG1AAG ICIs to human Fcγ receptors. The interaction between the anti‐Polyhistidine antibody and the His‐tagged receptors was not as stable as the interaction between Protein A and the IgG1AAG antibodies. Therefore, receptor binding at a certain timepoint (expressed as Response Units or RUs) was used to calculate the relative response of the antibodies to the Fcγ receptors, compared to IgG1k binding to the same receptor.

The ICIs should be ‘effector‐silent’ to minimize unwanted immune effector functions (Chen *et al*., 2019). Therefore, binding to activating receptors like FcγRI, FcγRIIa and FcγRIIIa is not desirable. For FcγRI, interestingly we found no significant differences between receptor binding of either mammalian cell‐ or plant‐made IgG4P ICIs, and IgG1 or IgG4 with non‐modified Fc regions (Tables 3B and S3A). The binding of IgG1AAG ICIs made in plants with WT or modified glycosylation (NtFX‐KO) were significantly lower than antibodies with non‐modified Fc regions (IgG1 and IgG4), as well as IgG4P ICIs made in mammalian cells or NtFX‐KO plants (*P* < 0.05; IgG1AAG NtFX‐KO and WT vs. IgG4 *P* = 0.0546 and 0.0577, respectively; Tables 3B and S3A). No significant FcγRI binding differences were seen between the two plant‐made IgG1AAG ICIs.

The binding of all IgG4P and IgG1AAG ICIs to FcγRIIa was significantly lower compared to IgG1 (Tables 3B and S3B). No significant differences were seen in FcγRIIa binding between all ICIs compared to IgG4, except for IgG4P produced in NtFX‐KO plants, which were significantly lower (Tables 3B and S3B). There were no significant differences between all other plant‐produced ICIs and mammalian cell‐produced IgG4P. However, it has to be noted that IgG4P produced in WT plants, as well as the two plant‐produced IgG1AAG, were borderline significantly lower (0.09 < *P* < 0.05). This may be regarded as significant if accounting for the relatively unstable interaction during the receptor capture step.

When comparing the two IgG4P ICIs with different glycosylation patterns, binding of IgG4P produced in WT plants to FcγRIIa was significantly lower than IgG4P NtFX‐KO (Tables 3B and S3B). However, there were no significant differences in receptor binding between the two plant IgG1AAG ICIs. Furthermore, we also found that plant IgG1AAG ICIs have significantly lower receptor binding compared to plant IgG4P ICIs.

For FcγRIIb/c, there were no significant differences between receptor binding of IgG4P ICIs with IgG1 and IgG4, even though there were several probability values that were borderline significant (Tables 3B and S3C). The exception is IgG4P with WT glycosylation, which has significantly lower binding. The plant IgG1AAG ICIs also had significantly lower FcγRIIb/c binding compared to IgG4P produced in mammalian cells and NtFX‐KO plants, as well as the IgG1 and IgG4 controls. Interestingly, there were no significant differences in receptor binding between WT IgG4P and the two IgG1AAG ICIs (Tables 3B and S3C).

For FcγRIIIa, the binding of all mammalian cell‐ and plant‐made ICIs were significantly lower than IgG1 (Tables 3B and S3D). Removal of plant α1,3‐fucose and β1,2‐xylose in the IgG4P glycan significantly increased FcγRIIIa binding compared to IgG4, mammalian cell‐made IgG4P and plant‐made IgG1AAG ICIs. In contrast, glycomodification of IgG1AAG ICIs did not alter receptor binding, with no significant differences seen between ICIs made in WT or NtFX‐KO plants, or between both IgG1AAG ICIs and all IgG4 antibodies or IgG4P ICIs (Tables 3B and S3D).

Based on the preliminary screening, IgG1AAG ICIs were brought forward for binding kinetics analyses. *S. aureus* Protein A capture format (Table S1) was used to determine the binding kinetics of all IgG1AAG ICIs to Fcγ receptors. There were differences in the binding curves of both IgG1AAG ICIs glycoforms to both FcγRI and FcγRIIIa, compared to IgG1 from mammalian cells and NtFX‐KO plants (Figures S5 and S6, respectively). The IgG1 antibodies also achieved target receptor binding (*R*_max_) within the concentration ranges of the experiment, while the IgG1AAG ICIs did not (Table S2C,F). However, within the population of IgG1AAG that bound to the receptor, it seems that there were no significant differences in the equilibrium dissociation constant (*K*_*D*_), ranging from 10^−7^ to 10^−9^ M compared to 10^−9^ to 10^−11^ M for the two IgG1s (Table S2C,F). For FcγRIIa, no binding was detected for either of the two plant‐made IgG1AAG ICIs (Table S2D), while for FcγRIIb/c, receptor binding was detected for IgG1AAG WT only (Table S2E).

### Binding of plant‐made ICIs to hC1q

ELISA was used to determine the binding of IgG4P and IgG1AAG ICIs made in plants (with WT and modified glycosylation) to hC1q. This was then compared to their mammalian cell‐made IgG4, IgG1, IgG4P and IgG1AAG counterparts. We found that IgG4P and IgG1AAG ICIs bound less well to hC1q compared to IgG4 or IgG1 with unmutated Fc regions, regardless of manufacturing platform (Figure S7). Furthermore, there were significant differences in hC1q binding between IgG4P and IgG1AAG ICIs made in WT plants compared to those made in NtFX‐KO plants (Figure S7). hC1q binding of all IgG4P ICIs made in mammalian cells and glycomodified plants were similar, but IgG4P ICI with WT plant glycosylation bound less. For IgG1AAG, the mammalian cell and WT plant versions bound slightly less to hC1q compared to NtFX‐KO ones. On the contrary, there were no significant differences in hC1q binding between IgG4P and IgG1AAG antibodies made in plants with the same glycosylation pattern (*i.e*. WT or NtFX‐KO; Figure S7).

### Production cost model

It is generally difficult to build holistic models for production costs that include development costs, account depreciation and sales margins. This is because these economic aspects are highly product dependent and often not disclosed (*e.g*. by companies).

Therefore, we have augmented our previous cost model (Buyel and Fischer, 2012; Fischer and Buyel, 2020; Knödler *et al*., 2023) with various options in upstream plant production and downstream biomass processing (Table S4). Two general production scenarios were derived: (1) fully‐automated transient expression using a vertical farm in a middle‐ to high‐income country for the production of emergency or personalized medicines (automated/transient; *e.g*. Ward *et al*., 2021); and (2) manual greenhouse cultivation of transgenic plants in a low‐ to middle‐income country (manual/transgenic; *e.g*. Ma *et al*., 2015).

Firstly, we defined vectors storing the ranges of product quantity, biomass per plant, protein accumulation in that biomass, and process recovery to be investigated (Equation 1).

(1) $$x_{k}=\left(x_{1,k}\ldots x_{n,k}\right)$$

Where x is vector storing the values, k is the parameter to be investigated (*e.g*. product quantity *q*), $x_{1,k}$ is the lower bound, and $x_{n,k}$ the upper bound of the respective parameter (Table S4). The step width that defined the number of vector elements can be selected freely—here, we have used step widths of 2 kg, 0.005 kg, 0.005 g/kg and 0.01 for the product quantity (q), biomass per plant (b), accumulation (a) and product recovery (r), respectively.

We have combined the four vectors to form a four‐dimensional matrix X of size 10 × 40 × 400 × 95 (q × b × a × r), representing a total of 15.2 × 10^6^ individual process performance conditions, and calculated the number of plants required (n, Equation 2) and total biomass (m, Equation 3) to be processed for each of these conditions.

(2) $$n\left(q,b,a,r\right)=\frac{q\times s}{b\times a\times r}$$

Where s is a safety factor, here 1.05, *i.e*. 5%.

(3) $$m\left(q,a,r\right)=\frac{q\times s}{a\times r}=n\times b$$

Next, we defined a matrix A storing the information for the process to be implemented (Equation 4).

(4) $$A=\left[\begin{array}{ccc} a_{1,1} & \cdots & a_{1,j}\\ \vdots & \ddots & \vdots \\ a_{1,i} & \cdots & a_{i,j} \end{array}\right]$$

Where $a_{i,j}$ are the matrix elements that define which setting to use at process step i for scenario j (*e.g*. the batch size in terms of plants). Here, a step refers to a distinct position in the process, *e.g*. extraction, clarification, purification. Where appropriate, a matrix element $a_{i,j}$ was implemented as a vector—*e.g*. to store information about individual consumable costs for infiltration or clarification as well as labour time. For example, the vector would hold values for all types of consumables such as silicone tubing (~€1.16 m^−1^), single‐use storage bags (€177.94 per piece) and connectors (~€0.74–€2.94 per piece depending on diameter and type).

The number of scenarios to be investigated can be arbitrarily large, limited only by the computational power of the hardware used for calculation. Here, we focused our investigation on two settings—one for LMICs (greenhouse, one‐step purification) and one using an automated facility (vertical farm, two‐stage purification).

Then, we calculated the number of production lots (*i.e*. batches, l, Equation 5) based on the conditions in X and settings in A.

(5) $$l\left(A,X\right)=\frac{m}{c}$$

Where c is the number of plants per lot (2000 or 6000 in our case).

We used this information to derive the required costs for each of the conditions in X and settings in A based on, *e.g*., tabulated consumable costs established from a total of 12 technical and 6 GMP manufacturing batches (Ma *et al*., 2015), as well as an exponential model of plant growth calibrated to the specific greenhouse and vertical farm setting we used (Huebbers and Buyel, 2021). For example, the latter was used to calculate the space requirements, labour time, as well as energy and fertilizer consumption depending on the plant size and cultivation duration. This approach is similar to others such as Mir‐Artigues *et al*. (2019).

**Figure 2:**
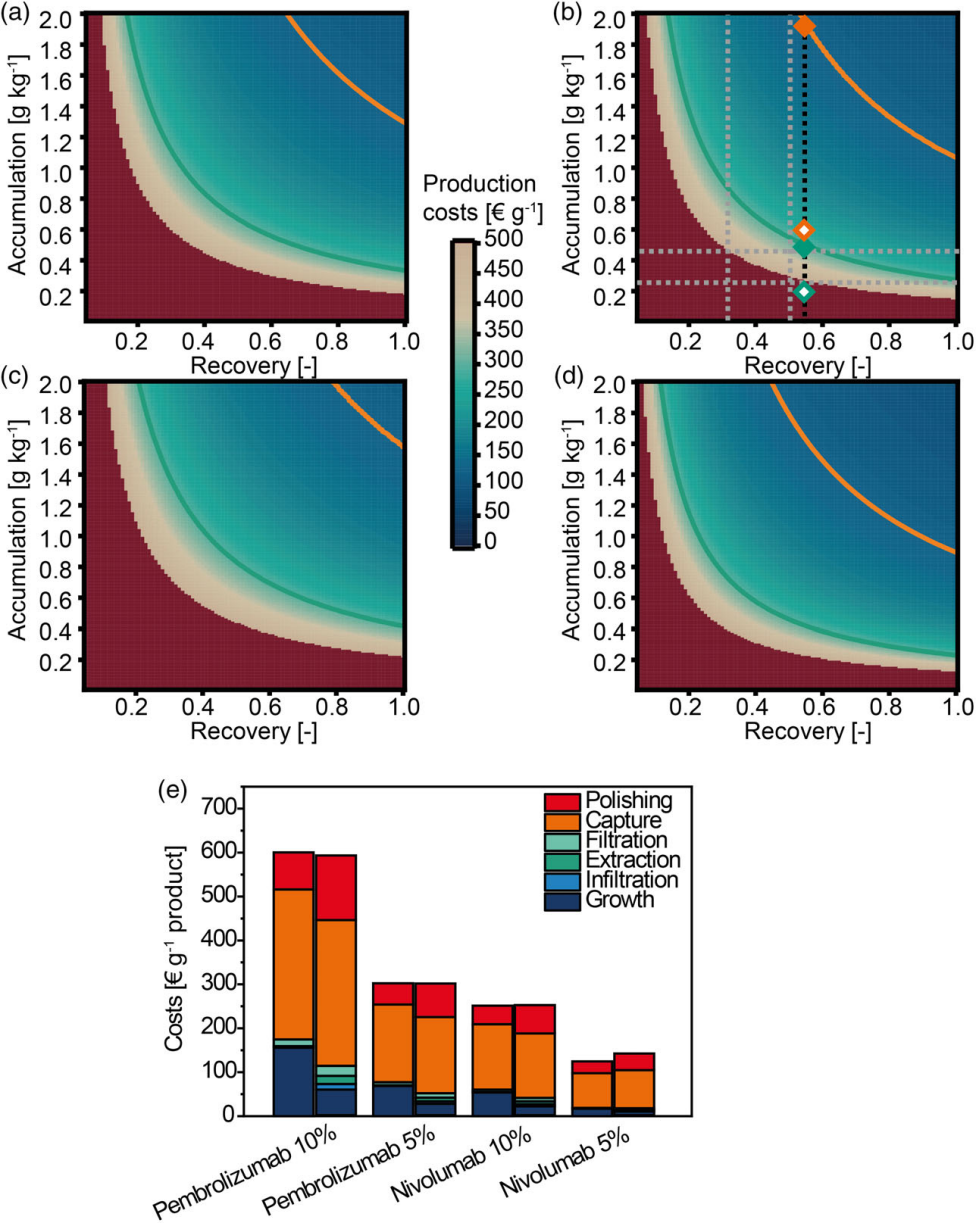
Cost heat‐maps for ICI production in plants based on accumulation level and recovery. Scenario for a low‐income country using transgenic plants in a greenhouse (manual/transgenic; a = 2000 plants; b = 6000 plants); and scenario for a high‐income country using *Agrobacterium tumefaciens*‐mediated transient expression in a vertical farm setting (automated/transient; c = 2000 plants; d = 6000 plants). Green and orange curves indicate the 5% sales price (as of 2019) Pareto front of ICIs Pembrolizumab (£5300 g^−1^ or €6000 g^−1^) and Nivolumab (£2200 g^−1^ or €2500 g^−1^), respectively. Grey horizontal lines indicate current accumulation in plants. Vertical lines indicate the current (grey, ~0.35 for IgG1AAG, ~0.50 for IgG4P; Table 4) and the prospected (black, 0.53) overall recoveries (based on Ma *et al*., 2015). Open and closed diamonds in b indicate spots where the plant‐based cost of goods is 10% and 5% of the 2019 product sales prices, respectively, at this overall recovery. (e) Cost type analysis for the manual/transgenic (left columns) and automated/transient (right columns) settings, identified in panels b and d, respectively (6000 plants per batch), for the 5% sales price condition of Nivolumab. Production costs for ICIs are segmented into growth, infiltration, extraction, filtration, capture and polishing.

The parameter settings like individual consumables costs or biomass per plant at the time of harvest were selected to represent typical process conditions. For example, the 0.15 kg and 0.35 kg wet biomass values were based on our typical purification recovery from *N. benthamiana* and *N. tabacum*, respectively. Apart from these settings, it was determined that product costs were largely governed by batch size, product accumulation and recovery. The latter two parameters can be regarded as indicators of upstream production and downstream processing performance, respectively. ‘Slices’ were extracted from the multi‐dimensional parameter space of the accumulation vs. recovery model to illustrate the dependency of product costs on these indicators (Figure 2a–d). The scales of the axes were chosen according to levels reported (up to ~2 g/kg) for transient monoclonal antibody production in *N. benthamiana* (Zischewski *et al*., 2016) and technically feasible recovery ranges. The calculations were conducted for batch sizes of 2000 and 6000 plants, which in our setting corresponded up to 700 and 2100 kg of biomass, respectively. Given that 1 kg of wet plant biomass is approximately equivalent to one litre of animal cell culture in terms of dry cell mass (Gengenbach *et al*., 2019; Sheen, 1983; Széliová *et al*., 2020), these batch sizes were comparable to the 2000‐L single‐use bioreactors used for the production of monoclonal antibodies, *e.g*. in CHO cells (Jacquemart *et al*., 2016).

The corresponding plots can be used to obtain an idea about production costs in a product‐independent manner. In our case, we added iso‐cost lines to the plots indicating the 5% sales prices Pareto front of Pembrolizumab (tradename KEYTRUDA®; 2019 sales price £5300 g^−1^ or €6000 g^−1^, green iso‐cost line) and Nivolumab (tradename OPDIVO®; 2019 sales price £2200 g^−1^ or €2500 g^−1^; orange iso‐cost line, Figure 2a–d). As a comparison, current sales prices of ICIs Pembrolizumab and Nivolumab are £26 300 g^−1^ (~€31 600 g^−1^; National Institute for Health Care and Excellence, 2021) and $28 895 g^−1^ (~€25 500 g^−1^; Centers for Medicare and Medicaid Services (CMS), 2021) as of August 2021. We then used the accumulation of ~0.46 g/kg (IgG1AAG) and ~0.23 g/kg (IgG4P) achieved by transient expression in *N. benthamiana* (Table 2) in combination with the respective recoveries of ~0.35 [−] and ~0.50 [−] to establish a current cost region for ICI production in plants (area inside the dashed grey lines in Figure 2b as a representative). The production costs calculated in this region were €400–€2000 g^−1^, and thus about 15–75‐fold lower than the 2021 sales prices of the competing mammalian cell‐derived ICIs.

We also wanted to consider development costs, account depreciation, and sales margins. As these economic aspects are strongly product‐dependent and are difficult to foresee, we assumed two potential breakeven options between the two commercialized mammalian cell‐based products and the plant‐derived ICIs we developed here: the cost of goods of plant‐produced ICIs were 10% (open symbols) or 5% (closed symbols, in Figure 2b) of the 2019 sales price of mammalian cell‐made ICIs, as a conservative estimate compared to the substantially higher 2021 sales prices. The cost values obtained for the breakeven options were €125–€300 g^−1^ and thus appeared realistic as the cost of goods for monoclonal antibody production in mammalian cells has been reported to be ~€100 g^−1^ (Yang *et al*., 2019), which corresponded to ~1.6% and 4.0% of the Nivolumab and Pembrolizumab 2019 sales prices we used in our calculation. An overall recovery of 53% was used for this calculation, which has been achieved for a fully developed GMP‐compliant process with >97% purity (Ma *et al*., 2015).

The accumulation levels of plant‐derived ICIs necessary to reach the 10% and 5% breakeven points were 0.23–0.80 g/kg (10%) and 0.52–2.23 g/kg (5%), respectively, in the manual/transgenic scenario, and similar for the automated/transient setting (Table 4). Therefore, all conditions for Pembrolizumab fell within the cost region of the ICIs we expressed in plants given a recovery of 0.53, while the production of Nivolumab was well within reach. Only the 5% breakeven condition of Nivolumab would require a threefold increase in protein accumulation level. This seems well in reach given reports of improvements in protein accumulation for plant‐produced antibodies ranged from ~0.5 g/kg (similar to the levels reported here) to ~2.5 g/kg (Zischewski *et al*., 2016).

**Table 4:** Required accumulation levels of ICIs in plants to achieve a breakeven situation with mammalian cell‐derived counterparts. Projections based on 2021 sales prices of ICIs Pembrolizumab £26 300 g^−1^ (~€31 600 g^−1^) and Nivolumab $28 895 g^−1^ (~€25 500 g^−1^)

Antibody	Pembrolizumab		Nivolumab	
Scenario (production costs as per cent 2021 sales price)	10% (€12 000 g^−1^)	5% (€6000 g^−1^)	10% (€5000 g^−1^)	5% (€2500 g^−1^)
	Required accumulation (g/kg LFW)			
Manual/transgenic (low income, 6000 plants per batch)	0.23	0.52	0.66	2.21
Automated/transient (high income, 6000 plants per batch)	0.28	0.63	0.80	2.23

## Discussion

ICIs are revolutionary in cancer treatments (Tang *et al*., 2018), but efforts have to be made to improve accessibility, especially to LMICs, by lowering costs. Combination therapy to improve their efficacy (Khair *et al*., 2019; Ma *et al*., 2021) also necessitates separate manufacturing lines for each component, which can increase manufacturing costs. We believe ICIs can be made more economically feasible using plants, a relatively new but attractive alternative production platform to the current mammalian cell‐based bioreactors (Moon *et al*., 2020; Paul *et al*., 2013).

Plants are capable of heterologous expression of complex proteins with multiple subunits or components, such as virus‐like particles (Ponndorf *et al*., 2021; Ward *et al*., 2021), and antibodies like secretory IgAs (Teh *et al*., 2021) and IgGs (Pinneh *et al*., 2022; van Dolleweerd *et al*., 2014). Here, we showed that three intact and fully functioning ICIs ‐ Nivolumab (anti‐PD‐1), Monalizumab (anti‐NKG2A) and Relatimab (anti‐LAG‐3)—can be expressed transiently in *N. benthamiana* and *N. tabacum* plants. The ICIs were expressed with two different Fc regions (IgG4P and IgG1AAG), and we have obtained protein accumulation levels of up to 0.77 g/kg LFW when co‐expressed with TBSV P19 silencing suppressor, all without compromising specificity to their target ligands. Rattanapisit *et al*. (2019) reported transient expression of Nivolumab in *N. benthamiana* at 0.14 g/kg LFW without P19 co‐expression, while Phakham *et al*. (2021) reported accumulation of Pembrolizumab, another anti‐PD1 ICI, of around 0.350 g/kg LFW. Although we did not compare the accumulation levels of transiently infiltrated and transgenic plants, previous studies have shown that there were no significant differences in accumulation levels of an IgG1k anti‐HIV‐1 antibody VRC01 (Teh *et al*., 2014). Therefore, we predict the protein accumulation levels of the ICIs to be similar to those without P19 co‐expression using the transient expression method.

Furthermore, there were no significant differences in protein accumulation levels between the IgG4P and IgG1AAG formats. However, the IgG1AAG format was found to improve purification recovery, especially at 100 mg‐scale, as IgG4P bound less well to Protein A compared to non‐Fc modified IgG4. Although we can use anti‐kappa or anti‐Fab binders, they are more expensive. At the kg‐scale, although using the IgG1AAG constant region increased the step recovery compared to IgG4P, the overall protein recovery decreased by about 10%. The higher loss may be due to the differences in extraction and clarification methods between 100 mg‐ and kg‐scale, an effect of protein aggregation, or interaction with process equipment as substantial quantities of product were lost during depth filtration. Using alternative filters, *e.g*. without diatomaceous earth, might help to mitigate this challenge in the future (Buyel *et al*., 2015).

Plant‐produced proteins have a more uniform glycosylation and this contributes to the overall consistency of the final product. Rattanapisit *et al*. (2019) have shown that Nivolumab produced in afucosylated and axylosylated *N. benthamiana* plants contained mainly GnGn without core plant α1,3‐fucose and β1,2‐xylose. Göritzer *et al*. (2022) also reported GnGn as one of the main glycoforms in VRC01 produced in NtFX‐KO plants.

Here, we showed that core α1,3‐fucose and β1,2‐xylose were present in ICIs expressed in WT plants and absent in ICIs produced in glycomodified NtFX‐KO plants. Neither the glycosylation pattern nor the Fc region had any impact on the binding to hFcRn at acidic pH (pH 6.0), or their ‘release’ at physiological pH (pH 7.4). This is in contrast to Stelter *et al*. (2020) who reported that WT glycosylation of non‐Fc modified IgG1 anti‐HIV antibodies affected hFcRn binding at pH 6. Further transcytosis experiments using cells stably expressing hFcRn can be performed to determine hFcRn recycling (Jaramillo *et al*., 2017) of ICIs, while half‐life experiments with transgenic mice expressing hFcRn (Petkova *et al*., 2006) can be used to determine the half‐lives of the ICIs.

For ICIs targeting immune cells such as Nivolumab, Monalizumab and Relatimab, binding to Fc receptors is not desirable to prevent unwanted effector functions (Chen *et al*., 2019). We have found that although ICIs with IgG4P Fc region have lower Fcγ receptor and hC1q binding than IgG1k, they were not as ‘effector‐silent’ as ICIs with IgG1AAG. The only Fcγ receptor that bound better to IgG4P was FcγRIIb/c, which may play a role in suppressing tumour‐infiltrating CD8^+^ T cells (Farley *et al*., 2021). Furthermore, glycosylation profile had an impact on IgG4P ICIs–ICIs with core plant α1,3‐fucose and β1,2‐xylose having lower affinity to FcγRIIa, FcγRIIb/c and FcγRIIIa.

In contrast, glycosylation had no measurable impact on IgG1AAG‐based ICIs. This might be due to the already low affinity to Fcγ receptor that these ICIs have. However, there was a small subset of IgG1AAG ICIs that bound to FcγRI and FcγRIIIa with high affinity. These were most probably ICIs with high mannose glycans that have previously been showed to have increased Fcγ receptor binding and increased antibody‐dependent cellular cytotoxicity (ADCC; Stelter *et al*., 2020; Zhou *et al*., 2008). Even though they were not the most abundant glycoform, high mannose glycoforms have been found in antibodies produced in *N. benthamiana* and *N. tabacum* plants with WT and modified glycosylation (Göritzer *et al*., 2022; Teh *et al*., 2014).

For hC1q, both IgG4P and IgG1AAG ICIs bound less well to the complement protein, with IgG1AAG less than IgG4P. Glycosylation profiles also made an impact on complement binding with the presence of core plant α1,3‐fucose and β1,2‐xylose negatively impacting the affinity to hC1q.

These results suggested that apart from amino acid mutations in the Fc region, glycosylation can also be used to fine‐tune affinities to different Fcγ receptors or completely abolish them without affecting ICI half‐life. Rattanapisit *et al*. (2019) demonstrated *in vitro* that apart from promoting T cells responses, their version of axylosylated and afucosylated Nivolumab also activates the NFAT pathway. On the contrary, Stelter *et al*. (2020) have found that VRC01 (an IgG1 which has an unmutated Fc region) containing plant α1,3‐fucose and β1,2‐xylose had less affinity to Fcγ receptors, especially FcγRIIIa, compared to VRC01 with the mammalian α1,6‐fucose. This suggested ICIs containing plant glycans might be the more suitable ‘effector silent’ candidate.

Plant glycans have been linked with an increased risk for immunogenicity and adverse allergic reactions (Bardor *et al*., 2003; Jin *et al*., 2008; Paulus *et al*., 2011). However, the significance of these risks has not been established (McCormick *et al*., 2003; Shaaltiel and Tekoah, 2016), and plant‐made therapeutics with WT glycosylation have been consistently demonstrated to be safe for human use (Ma *et al*., 2015; Zimran *et al*., 2018). Furthermore, there is evidence that the ‘adjuvanticity’ of an immunotherapeutic is an advantage (Bai *et al*., 2020; Seya *et al*., 2018)—plant glycans may have an impact on the local tumour microenvironment because the immunogenicity stimulates the activity of APCs (*e.g. via* lectin or mannose/fucose receptors on DC; Rosales‐Mendoza *et al*., 2016).

The production cost model indicated that the manual/transgenic setting (Figure 2; a + b) does profit less from an increased batch size (2000 in A; 6000 in B), compared to an automated/transient setup (Figure 2; c + d). Specifically, in the latter at a batch size of 2000 plants (Figure 2c), a production at 5% of the 2019 sales prices cannot be met at 60% recovery at any ICI accumulation levels of <2 g/kg. However, ~1.3 g/kg is sufficient to meet this criterion for a batch size of 6000 (Figure 2d). Furthermore, growth costs were 100% more for the manual/transgenic setting compared to the automated/transient setting. This difference is offset by increased polishing costs due to a second purification step included in the automated/transient setting.

In this context, the downstream processing can be simplified in the future to further improve the economic viability of plant‐derived ICIs. In the cost of goods breakdown, capture and polishing chromatography were the major cost drivers accounting for 69%–81% of the total production costs. This can be improved through membrane‐based purification, or combined pH and temperature pre‐treatment as shown for ICIs and other recombinant antibodies or proteins produced in plants (Opdensteinen *et al*., 2019, 2021).

Furthermore, we found that there is an economy‐of‐scale for plant‐based processes too, mostly due to fixed costs accounting for a smaller fraction of the total, and some costs scale sub‐linearly, *e.g*. buffer preparation, cleaning *etc*. Therefore, a breakeven in terms of cost of goods with mammalian cell culture‐based expression systems may also be achieved by scaling the process up instead of increasing product accumulation. Assuming a treatment with 7.5 mg/kg body mass (Jiang *et al*., 2022), which is common for ICIs, and an average body mass of 70.8 kg for Europeans (Walpole *et al*., 2012), a treatment period of a year with interventions every three weeks amounts to 9.2 g of ICI per patient. Given that there are about 400 000 new cancer patients per year in medium‐sized industrialized countries (*e.g*. UK) and LMIC (*e.g*. Indonesia; World Cancer Research Fund International, 2022), of which about 12% can be expected to respond to ICI treatment (Haslam *et al*., 2020), ~48 000 patients will require ICIs which amounts to ~450 kg/a in such countries.

This implies that our current production process has to be scaled up by a factor of at least 23 compared to our current setting of 20 kg of purified product, assuming local production resilience towards supply chain uncertainty. Applying this factor in a typical empirical formula to estimate the economy of scale (Equation 6; Moore, 1959), and using a scaling coefficient s of ~0.65 reported for plant processing equipment (Tribe and Alpine, 1986), one can expect the capital costs to increase about 7.5‐fold (6.5‐fold for s = 0.60 and 8.8‐fold for s = 0.70), which corresponds to a 70% reduction relative to the production capacity.

(6) $$C_{2}=C_{1}{\left(\frac{n_{2}}{n_{1}}\right)}^{s}$$

Where $C_{2}$ is the cost of the scaled‐up equipment, $C_{1}$ is the equipment cost at the current scale, $n_{2}$ and $n_{1}$ are the capacities of the scaled‐up and current process, respectively, and s is the scaling coefficient.

A similar scaling approach has been previously used for other plant‐based manufacturing processes (Nandi *et al*., 2016). However, the reported correlation between capital costs and process scale is typically only one order of magnitude. Furthermore, the authors assumed that the same type of processes and equipment will be used in the scaled‐up production. Both scenarios are unlikely to be applied in this case due to the substantial increase in scale, the anticipated changes in process design (*e.g*. the use of UF/DF and continuous processes), as well as the prototypical nature of most pilot and production scale facilities for GMP‐compliant plant molecular farming.

Having said that, our findings are in agreement with what others have reported in terms of costs for the plant‐based production of non‐antibody protein Griffithsin (Alam *et al*., 2018). Finally, it is important to note that, in addition to the conventional cost of goods, life cycle assessment accounting for the costs of the environmental footprint of manufacturing is becoming increasingly important, even for biopharmaceutical manufacturing (Amasawa *et al*., 2021). In this context, we believe plants can have substantial benefits as they can be regarded as self‐building, single‐use, biodegradable bioreactors.

To conclude, we have demonstrated that fully functioning ICIs can be made using the plant manufacturing platform. Changing the Fc region as well as the glycosylation profiles of the ICIs can alter their protein recovery and their binding to Fcγ receptors without affecting hFcRn binding. We have also used a scenario‐based production cost model to show that the protein accumulation and recovery levels are as competitive as mammalian cell‐based production platforms. This highlights the potential to deliver ICIs that are more affordable and accessible to a more widespread market, including LMICs. In the future, a more detailed analysis of the impact of fluctuations in consumables costs, *e.g*. due to inflation or supply chain shortages, will help to improve our understanding about the robustness of the commercial viability of plant‐based production systems.

## Methods

### Construction of ICIs and cloning into MIDAS‐P expression vectors

The sequence of the heavy and light variable region (*V*_H_ and *V*_L_, respectively) of anti‐NKG2A ICI Monalizumab (Van Hall *et al*., 2019), anti‐PD‐1 Nivolumab (Topalian *et al*., 2012), and anti‐LAG‐3 Relatimab (Ascierto *et al*., 2017) were obtained from the DrugBank database (Wishart *et al*., 2017). The *V*_H_ and *V*_L_ were plant‐codon optimized (GeneArt, ThermoFisher, Waltham, Massachusetts), domesticated to remove NcoI, XbaI, BsmBI and BsaI sites, synthesized with flanking BsaI sites, and cloned into pDONR‐Zeo backbone containing the constant region of human IgG4 with a S228P mutation (IgG4P; Silva *et al*., 2015) and human kappa chain, respectively. The *V*_H_ of Nivolumab were also cloned into a human IgG1 constant region with a L324A/L325A/P329G mutation (IgG1AAG; Schlothauer *et al*., 2016). The heavy and light chains were then cloned into the pWHITE and pBLUE modules of MIDAS‐P (Pinneh *et al*., 2022), respectively, using flanking NcoI and XbaI (NEB, Ipswich, Massachusetts) restriction sites. Then, they were sequentially ligated into the pMIDAS destination vector using BsaI for pWHITE or BsmBI for pBLUE. ICI heavy and light chain genes in MIDAS‐P vectors were then transformed into *Agrobacterium tumefaciencs* GV3101:: pMP90(RK) and grown on Yeast Extract Mannitol (YEM) medium (0.04% Yeast extract, 10 g/L^−^ mannitol, 1.7 mm NaCl, 0.8 mm MgSO_4_, 2.2 mm K_2_HPO_4_, pH7.0) with 1.5% (w/v) bacteriological agar, at 28 °C.

### Cloning of ICIs into mammalian cells expression vectors

Monalizumab and Relatimab IgG4P heavy chains were amplified with forward primer (5′ATCCTGCAGGATGGAACTTGGACTTTCT‐3′) and reverse primer (5′TATGATCATCATTACTTGCCAAGGCTCAG‐3′) containing SbfI and BclI, respectively, to generate complementary cohesive ends to PstI and BamHI. On the contrary, the light chains were amplified using forward (5′ATCTGCAGATGGACATGAGAGTTCCA‐3′) and reverse (5′TAGGATCCTCATTAGCACTCGCCCCTATT‐3′) primers containing PstI and BamHI, respectively. The heavy and light chains were ligated into separate gWIZ™ mammalian cell expression vector (Genlantis, San Diego, California) which were pre‐digested with PstI and BamHI (NEB, Ipswich, Massachusetts), and then treated with Antarctic Phosphatase. Ligated constructs were transformed into *E. coli* DH10B and plated on LB agar containing 50 μg/mL Kanamycin. Plasmids were isolated using QIAPrep Spin Miniprep Kit (Qiagen, Düsseldorf, Germany) for transfection.

### Transient expression of ICIs in plants

For 100 mg‐scale infiltration, *Agrobacterium tumefaciens* GV3101:: pMP90(RK) transformed with the relevant plant expression vectors were grown to the required volume at 28°C in LB broth (10 g/L tryptone, 5 g/L yeast extract, 10 g/L NaCl, pH 7) supplemented with 50 μg/mL Rifampicin, 50 μg/mL Kanamycin and 50 μg/mL Carbenicillin (all Apollo Scientific, Bredbury, Stockport, UK). The bacterial suspension was diluted with Infiltration Solution (IS) containing 0.01 mm MES (Sigma‐Aldrich, St. Louis, Missouri) pH 5.6, 0.01 mm MgCl_2_ (VWR International, Radnor, Pennsylvania) and 0.1 mm acetosyringone (Santa Cruz Biotechnology, Dallas, Texas) to the final infiltration OD_600_ of 0.2. Fully expanded leaves of 5 to 7‐week‐old *N. benthamiana* WT or 10‐week‐*old N. tabacum* β(1,2)‐xylosyltransferase and α(1,3)‐fucosyltransferase knockout plants (NtFX‐KO; Göritzer *et al*., 2022) grown in all‐purpose compost, at 25 °C under a 16 h light/8 h dark cycle at 80–100 μmol/m^2^/s, were either transformed using syringe‐mediated infiltration or vacuum infiltration (Kapila *et al*., 1997). The plants were then further grown for six days under the same conditions before harvesting.

For kg‐scale infiltration, *Agrobacterium* harbouring ICI constructs were cultured in PAM4 (Houdelet *et al*., 2017) using half the antibiotics concentrations stated above. They were also diluted to OD_600_ of 0.2 with IS before being used to transform 7‐weeks old WT *N. benthamiana*, grown under the conditions described above, by vacuum infiltration using a vacuum pressure of 10 kPa (0.1 bar). The plants were grown under the same conditions for six days before harvesting.

### Transient expression of ICIs in mammalian cells

The Expi293™ Expression System (ThermoFisher, Waltham, Massachusetts) was used for the production of mammalian cell‐made Monalizumab and Relatimab according to the manufacturer's protocols. Briefly, Expi293F cells were grown in Expi293™ Expression Medium in non‐baffled vent‐cap suspension cells culture flasks on a shaker at 130 rpm in a 37°C incubator with 80% relative humidity and 7% CO_2_. Cells with a density of 3–5 × 10^6^ cells/mL were split to a final density of 2.5–3 × 10^6^ cells/mL and allowed to grow overnight. Cells with a density of 4.5–5.5 × 10^6^ viable cells/mL and 95%–99% viability were used for transfection. 30 μg of plasmid DNA (15 μg each of heavy or light chain in gWIZ) were transfected into 30 mL of cells using ExpiFectamine™ 293 reagent diluted in Opti‐MEM™ I Reduced Serum Medium before being incubated in a 125 mL suspension cell culture flask. ExpiFectamine™ 293 Transfection Enhancers 1 and 2 were added 18 h post‐transfection and 5% of Penicillin/Streptomycin was added 24 h post‐transfection. Cell medium was harvested at 6 days post‐transfection.

### Protein extraction and purification

For proteins produced in plants at 100 μg‐scale and 100 mg‐scale, infiltrated leaves were homogenized with three volumes (300 μL to 100 mg LFW) of extraction buffer (50 mm phosphate buffer pH 8.0, 1 μm PMSF). 3.175 mm chrome steel ball bearings and a Mixer Mill MM400 (Retsch, Haan, Germany) were used for 100 μg‐scale, while a blender was for 100 mg‐scale. Plant debris was removed by centrifugation and passed through 0.4 μm and 0.25 μm filters for 100 mg‐scale. At kg‐scale, extraction was carried out as previously described with a blade‐based homogenizer (Buyel and Fischer, 2015). Briefly, three volumes of extraction buffer (50 mm sodium phosphate pH 8.0, 500 mm sodium chloride, 10 mm sodium bisulfite) were added per kg of biomass (*e.g*. 3 L per 1 kg), homogenized for 3 × 30 s with 30 s interspersed breaks. The extract was clarified using a combination of bag, depth (PDF4; ~0.5–10 μm) and sterile (0.2 μm) filters (Buyel and Fischer, 2014). For Expi293F‐made ICIs, cell medium was passed through 0.4 μm and 0.25 μm filters.

At 100 mg‐scale and for Expi293F‐made ICIs, the clarified crude extracts were purified using a Protein A‐Agarose (Sigma‐Aldrich, St. Louis, Missouri) column. The column was washed with 10 column volumes (CVs) of Binding buffer (50 mm sodium phosphate, pH 8.0) and the protein eluted with Elution buffer (0.1 m glycine‐HCl, pH 2.7). The eluted fraction was neutralized with 1 m Tris–HCl, pH 9.0. At kg‐scale, the clarified extract was loaded to a column packed with 46 mL (Cytiva, Marlborough, Massachusetts) MAbSelectSure™ (ThermoFisher, Waltham, Massachusetts) at 10 mL/min. After a 5 CVs wash step with equilibration buffer (50 mm sodium phosphate pH 8.0, 500 mm sodium chloride, 10 mm sodium bisulfite), a pH 3.0 wash step was carried out for 5 CVs before the product was eluted at pH 2.3 for 3 CVs. The elution fraction was immediately neutralized using 0.6 v/v 0.5 M disodium hydrogen phosphate, pH 9.0.

Purified protein fractions were dialysed into PBS, pH 7.6 and then concentrated using Amicon Ultra‐15 (Molecular cutoff 100 kDa; Merck Millipore, Darmstadt, Germany). Size‐exclusion chromatography (SEC) on a HiLoad 16/600 Superdex 200 pg column (GE Healthcare, Chicago, Illinois) connected to an Äkta pure FPLC system (GE Healthcare, Chicago, Illinois) pre‐equilibrated with PBS pH 7.8 (pH 8.0 for Monalizumab) was then used to separate out fully assembled proteins at ~150 kDa. The relevant fractions were dialysed into 0.1× PBS, pH 7.6 (pH 8.0 for Monalizumab) before freeze‐drying overnight at −40 °C, <0.2 mbar. For Nivolumab IgG4P and Relatimab IgG1AAG, no freeze‐drying was performed.

### PAGE and Western blot

Purified antibody concentration was measured at A_280_ with a Nanodrop 2000 (ThermoFisher, Waltham, Massachusetts). To determine protein integrity, purified ICIs or antibody standards were mixed with NuPAGE LDS sample buffer with or without β‐mercaptoethanol, boiled for 10 min at 100 °C, and resolved on NuPage 4%–12% Bis‐Tris gel with MOPS buffer (Invitrogen, Waltham, Massachusetts). Human IgG1k or IgG4k (both Sigma‐Aldrich, St. Louis, Missouri) were used as positive controls. 1–2 μg of protein was used for PAGE and the gels were stained with InstantBlue (Expedeon, Cambridge, UK).

Fifty nanograms of protein was used for Western blot and the gels were transferred to a nitro‐cellulose membrane for immunoblotting. The membrane was blocked with 5% w/v semi‐skimmed milk in Tris‐buffered saline supplemented with 0.1% v/v Tween 20 (TBS‐T) before being probed with either peroxidase‐conjugated polyclonal sheep anti‐human kappa light chain antibody (1 in 10 000; Binding site, Birmingham, UK), peroxidase‐conjugated sheep anti‐human IgG1 gamma chain (1 in 5000; Binding Site, Birmingham, UK), or peroxidase‐conjugated rabbit anti‐human IgG4 gamma chain (1 in 5000; Invitrogen, Waltham, Massachusetts). Protein band detection was done using the ECL Prime system (ThermoFisher Pierce, Waltham, Massachusetts) and was visualized using G:Box F3 (Syngene, Cambridge, UK).

### ELISA

For quantification of IgG1 or IgG1AAG ICIs, 96‐well plates were coated with 1 in 200 sheep anti‐human gamma 1 (The Binding Site, Birminghamm, UK) diluted in PBS for 2 h at 37 °C. Blocking was performed for 1 h at 37 °C with Blocking Solution (5% w/v milk in PBS + 1% v/v Tween 20) and crude plant extract or purified proteins diluted in Blocking Solution were incubated at 37 °C for 2 h. Between each step, the plates were washed with deionized water +0.1% Tween‐20. Detection was with 1 in 1000 peroxidase‐conjugated sheep anti‐kappa (The Binding Site, Birminghamm, UK).

For IgG4 or IgG4P ICIs, sheep anti‐human kappa (Binding Site, Birminghamm, UK) diluted 1 in 200 in PBS was used to coat plates. The plates were blocked and the sample was introduced as previously described. Detection was with 1 in 1000 peroxidase‐conjugated rabbit anti‐human gamma 4 chain (Invitrogen, Waltham, Massachusetts). 3,3′,5,5′‐tetramethylbenzidine (TMB) substrate solution (Sigma‐Aldrich, St. Louis, Missouri) was used to detect peroxidase‐bound antibodies. The colour reaction was then stopped by the addition of 2 M sulphuric acid and the absorbance was measured at 450 nm using the Infinite F200 Pro plate reader (TECAN, UK). Titrations of IgG1k (Sigma‐Aldrich, St. Louis, Missouri) or IgG4k (InvivoGen, San Diego, California) were used as standards to determine antibody concentration.

To determine binding to hC1q, plates were coated with 5 μg/mL hC1q (Sigma‐Aldrich, St. Louis, Missouri) overnight at 4 °C. The plates were washed and blocked as previously described. Samples were serially diluted in Blocking Solution and incubated at 4 °C overnight. A 1 in 1000 dilution of peroxidase‐conjugated anti‐human gamma (The Binding Site, Birminghamm, UK) in Blocking Solution was used for detection. Peroxidase‐bound antibody was detected and read as previously described.

### Surface plasmon resonance

Surface plasmon resonance was used to determine the binding kinetics of the ICIs to *S. aureus* Protein A, as well as to the different human Fcγ receptors and hFcRn. These were performed on BIAcore X100 (Cytiva, Marlborough, Massachusetts) at 25 °C. Unless stated otherwise, all experiments were performed with a CM5 chip coated on both flow cells with Protein A to 3000–4000 response units (RUs) using standard amine coupling, and HBS‐EP+ (10 mm HEPES, pH 7.4, 150 mm NaCl, 3 mm EDTA and 0.05% surfactant P‐20) as Running Buffer. Bound proteins were removed with two 60 s pulses of Regeneration Buffer (10 mm glycine‐HCl, pH 1.5). All experiments were conducted at 25 °C unless stated otherwise.

Antibody binding to Protein A was evaluated using a CM5 chip with 50 Reference Units (RUs) of BSA coated on flow cell 1, and 50 RUs of *S. aureus* Protein A (Sigma‐Aldrich, St. Louis, Missouri) coated on flow cell 2. 3.125 to 100 μg/mL of ICIs or antibody standards were injected at 30 μL/min for 120 s (association), followed by 3000 s of Running Buffer (dissociation). Bound proteins were removed by pulses of regeneration buffer.

Binding of IgG4P to human FcγRIa or FcγRIIIa was determined by using a CM5 chip coated with anti‐Polyhistidine‐tag antibodies to ~600 RU using a His Capture kit (Cytiva, Marlborough, Massachusetts). 5 μg/mL of Polyhistidine‐tagged FcγRIa or FcγRIIIa were captured on one flow cell for 100 s at 10 μL/min. Then, 100 μg/mL of ICIs or controls (concentration determined with Nanodrop) was flowed over both flow cells for 60 s, followed by 120 s of dissociation time at 30 μL/min. The chip was then regenerated with 10 mm glycine‐HCl, pH 3, for 30 s at 30 μL/min. Binding was determined at 5 s before the end of antibody injection. Startup cycles with running buffer were run at the beginning and end of each set of runs.

Binding of Ig1AAG ICIs to human Fcγ receptors (R&D Systems, NE Minneapolis, Minnesota) was determined by using a CM5 chip coated with ~3000 RU of *Staphylococcus aureus* Protein A (Sigma‐Aldrich, St. Louis, Missouri). The kinetics of antibodies to the Fc receptors were determined by parameters optimized previously (Stelter *et al*., 2020). Briefly, antibodies were captured to the required level on one flow cell. Then, Fcγ receptors were injected at multiple concentrations over both flow cells for a specified time and allowed to dissociate for another period of time (detailed in Table S1). The chip was regenerated with 10 mm glycine‐HCl, pH 1.5.

Binding of IgG1AAG ICIs to hFcRn (R&D Systems, NE Minneapolis, Minnesota) was determined using a CM5 chip coated with ~9000 RU of human Fab binder from the Human Fab Capture Kit (Cytiva, Marlborough, Massachusetts). PBS, pH 6, with 0.05% Tween‐20 was used as Running Buffer. Antibodies were captured to 345 RU on one flow cell. hFcRn were injected at 37.5–200 nm over both flow cells for 60 s and allowed to dissociate for 90 s. The chip was regenerated with 10 mm glycine‐HCl, pH 2.1.

All sensorgrams were corrected with appropriate blank references and fit globally with BIAcore Evaluation software using a 1:1 Langmuir model of binding unless stated otherwise.

### Immune cell isolation

Peripheral Blood Mononuclear Cells (PBMCs) were isolated from healthy donor volunteers of leukocyte reduction system (LRS) cones from the national health service blood transfusion unit at St George's Hospital, London. PBMC were isolated using density centrifugation over Histopaque‐1077 (Sigma‐Aldrich, St. Louis, Missouri) as per the manufacturer's instructions. CD3+ T‐cells were isolated from PBMC by positive selection using magnetic CD3+ T‐cell MicroBeads (Miltenyi Biotec, Bergisch Gladbach, Germany), according to the manufacturer's instructions. With the remaining CD3 negative cells, CD56+ cells were isolated using CD56+ MicroBeads (Miltenyi Biotec, Bergisch Gladbach, Germany). Isolated CD3+ T‐cells and CD56+ NK cells had consistent median purities of >90%.

### Culture of immune cells

Freshly isolated CD3+ T‐cells and CD56+ NK cells were cultured at 1 × 10^6^ cells/mL in RPMI 1640 + 10% FBS (both Sigma‐Aldrich, St. Louis, Missouri), in 200 μL total volume in 96 well flat bottom plates. T‐cells were stimulated with anti‐CD2/CD3/CD28 coated T‐cell activation beads (Miltenyi Biotec, Bergisch Gladbach, Germany) with a bead‐to‐cell ratio of 1:2 according to the manufacturer's instructions. NK cells were stimulated with 50 ng/mL IL‐15 (BioLegend, San Diego, California) and 100 ng/mL IL‐2 (R&D Systems, NE Minneapolis, Minnesota). All cells were cultured for 72 h at 37 °C with 5% CO_2_.

### Multiparameter flow cytometry

Cells were stained with Zombie Aqua viability dye (BioLegend, San Diego, California) prior to antibody staining. Staining was performed in FACS buffer (PBS containing 2.5% BSA, 0.1% sodium azide and 2 mm EDTA) for 30 min at 4 °C. Cells were stained sequentially, first with plant‐made ICIs against immune checkpoints NKG2A (Monalizumab), PD‐1 (Nivolumab) or LAG‐3 (Relatimab) with IgG4P or IgG1AAG constant regions, mammalian‐cell‐made IgG4P Monalizumab and Relatimab, commercial IgG4P Nivolumab (Absolute Antibody, Redcar, Cleveland, UK), non‐Fc mutated IgG1k (Sigma‐Aldrich, St. Louis, Missouri) or IgG4k controls (InvivoGen, San Diego, California). Cells were then stained with secondary antibody anti‐IgG‐FITC (Sigma‐Aldrich, St. Louis, Missouri). Finally, cells were stained with primary conjugated antibodies—for T‐cells, antibodies included CD3‐BUV395, (BD Biosciences, San Jose, California), CD4‐PerCPCy5.5, CD8‐BV421, CD45RA‐APC and CCR7‐PECy7 (BioLegend, San Diego, California); for NK cells antibodies included CD3‐BUV395 and CD56‐BUV737 (BD Biosciences, San Jose, California).

Data was collected on Fortessa X20 (BD Biosciences, San Jose, California) and analysed using FlowJo (Tree Star, Ashland, Oregon). Debris was excluded by SSC‐A versus SSC‐W and live cells were gated based on the exclusion of the viability dye. T‐cells and NK cells were gated as CD3+ and CD56+, respectively, and further examined. The gating strategy for immune checkpoints on CD3+ T‐cells and CD56+ NK cells is detailed in Figure S8.

### Production cost model

An existing top‐down production cost model to calculate the costs for recombinant protein manufacturing in plants (Buyel and Fischer, 2012; Fischer and Buyel, 2020) was augmented as described in the results section, *e.g*. by introducing screw‐press extraction (Buyel and Fischer, 2015) and pre‐coat filtration process options (Buyel *et al*., 2015; Figure S9). The input parameter values and ranges for the model were selected based on a representative, plant‐based monoclonal antibody manufacturing process complying with GMP (Table S4; Ma *et al*., 2015). The model was implemented using the open‐source software GNU Octave version 7.1.0.

### Statistics

Normality of all data was tested and the null hypothesis was rejected if *P* < 0.05 using the Shapiro–Wilk test; if found to be normal, a one‐way ANOVA test was carried out. If data was significantly skewed, a non‐parametric Kruskal–Wallis test was used for data analysis. The homogeneity of variance was also tested for each sample data set using the Levene test and the null hypothesis was rejected if *P* < 0.05, resulting in the analysis of a data set either with the Brown‐Forsythe and Welch correction or ordinary one‐way ANOVA test depending on Levene test outcome. *Post hoc* analysis of the different sample groups was carried out either using the Dunn Bonferroni or the Tamhane T2 *post hoc* multiple comparison test depending on the outcome of the Levene test. All graphs were drawn and analysed using the GraphPad prism 8 software (GraphPad, San Diego, California).

## Conflict of interest

The authors declared no conflict of interest.

## Author contributions

LR, MBS, JB and AT provided substantial contributions to the conception of the work. All authors substantially contributed to the acquisition, analysis or interpretation of data for the manuscript and drafting, revising and critically reviewing the manuscript for important intellectual content.

## Supporting information


**Table S1** SPR experimental parameters for human Fcγ Receptor binding to IgG1AAG ICIs.
**Table S2** Binding of IgG1AAG ICIs to hRcFn and human Fcγ receptors.
**Table S3** Probability (p) values of Fcγ receptor binding differences of antibodies and ICIs.
**Table S4** Input parameter settings used for the calculation of production costs of plant‐derived ICIs.
**Figure S1** PAGE gel of purified ICIs.
**Figure S2** Western blot of purified ICIs.
**Figure S3** Glycosylation of ICIs.
**Figure S4** Binding of ICIs to *S. aureus* Protein A.
**Figure S5** Binding of antibodies and ICIs to human FcγRI receptor.
**Figure S6** Binding of antibodies and ICIs to human FcγRIIIa receptor.
**Figure S7** Binding of ICIs to hC1q.
**Figure S8** Gating strategy for PD‐1, LAG‐3, NKG2A expression.
**Figure S9** Top‐down model workflow to calculate the production costs per gram of ICI.
