# Supplementary material for: Killer to cure: Expression and production costs calculation of tobacco plant‐made cancer‐immune checkpoint inhibitors
Source: Plant Biotechnol J. 2023 Mar 18;21(6):1254–69. doi: 10.1111/pbi.14034 (PMC10214761; doi:10.1111/pbi.14034)
Supplement: Supplementary file 1 — Table S1 SPR experimental parameters for human Fcγ Receptor binding to IgG1AAG ICIs. Table S2 Binding of IgG1AAG ICIs to hRcFn and human Fcγ receptors. Table S3 Probability (p) values of Fcγ receptor binding differences of antibodies and ICIs. Table S4 Input parameter settings used for the calculation of production costs of plant‐derived ICIs. Figure S1 PAGE gel of purified ICIs. Figure S2 Western blot of purified ICIs. Figure S3 Glycosylation of ICIs. Figure S4 Binding of ICIs to S. aureus Protein A. Figure S5 Binding of antibodies and ICIs to human FcγRI receptor. Figure S6 Binding of antibodies and ICIs to human FcγRIIIa receptor. Figure S7 Binding of ICIs to hC1q. Figure S8 Gating strategy for PD‐1, LAG‐3, NKG2A expression. Figure S9 Top‐down model workflow to calculate the production costs per gram of ICI. [file PBI-21-1254-s001.docx]

**Table S1. SPR experimental parameters** **for human Fcγ Receptor binding to IgG1AAG ICIs**. CM5 chip coated with ~2000RU *S. aureus* Protein A (Sigma-Aldrich, USA) and HBS-EP+ (10 mM HEPES, pH 7.4, 150 mM NaCl, 3 mM EDTA and 0.05% surfactant P-20) was used as Running Buffer. Chip surfaces were regenerated with 2 pulses of 10 mM glycine-HCl, pH 1.5.

| Receptor | Temperature | Ligand | Ligand flow rate | Rmax | Receptor concentration | Contact time | Dissociate time | Flow rate |
| --- | --- | --- | --- | --- | --- | --- | --- | --- |
| FcγRI | 25^o^C | Individual mAbs/ICIs | 20 μL min^-1^ | 50 | 3.75 to 60 nM | 135 | 600 | 40 |
| FcγRIIa  (R167) | 37^o^C | Individual mAbs/ICIs | 25 μL min^-1^ | 100 | 0.1875 to 1.5 uM | 30 | 60 | 45 |
| FcγRIIb | 37^o^C | Individual mAbs/ICIs | 25 μL min^-1^ | 100 | 0.1875 to 1.5 uM | 30 | 60 | 45 |
| FcγRIIIa  (V158) | 25^o^C | Individual mAbs/ICIs | 20 μL min^-1^ | 50 | 93.75 to 750 nM | 135 | 600 | 40 |

**Table S2. Binding of IgG1AAG ICIs to hFcRn and human Fcγ receptors.** (A) Binding of IgG1AAG- and IgG4P ICIs produced in plants with wild-type glycosylation (WT) or axylosylated and afucosylated glycans (NtFX-KO) to (A) hFcRn at pH 6.0; (B) hFcRn at pH 7.4; (C-F) Binding of IgG1AAG ICIs produced in mammalian cells or plants with wild-type glycosylation (WT) or axylosylated and afucosylated glycans (NtFX-KO) with (C) human FcγRI; (D) human FcγRIIa; (E) human FcγRIIb/c; (F) human FcγRIIIa. IgG1k with non-modified Fc region made in glycomodified plants (plant IgG1k) or IgG1k or IgG4k from human myeloma cells (IgG1k or IgG4k; Sigma, USA) were used as positive control. SPR experiments were conducted using parameters detailed in Table S1. Two biological repeats were conducted for each experiment.

**A
hFcRn pH 6.0 (2-state reaction model)**

| **Antibody/ICI** | | | | | **K_D_** | | **ka1 (1/Ms)** | | | | **SE(ka1)** | | | | | **kd1 (1/s)** | | | | | **SE(kd1)** | | | | **ka2 (1/s)** | | | **SE(ka2)** | | **kd2 (1/s)** | | **SE(kd2)** | |
| --- | --- | --- | --- | --- | --- | --- | --- | --- | --- | --- | --- | --- | --- | --- | --- | --- | --- | --- | --- | --- | --- | --- | --- | --- | --- | --- | --- | --- | --- | --- | --- | --- | --- |
| IgG1k MC | | | | | 2.55E-007 | | 1.56E+006 | | | | 3.10E+005 | | | | | 0.3963 | | | | | 8.80E-002 | | | | 1.02E-007 | | | 6.40E-007 | | 0.003093 | | 4.50E-003 | |
| IgG1k NtFX-KO | | | | | 5.03E-008 | | 1.00E+006 | | | | 3.10E+004 | | | | | 0.1007 | | | | | 0.0044 | | | | 0.01345 | | | 4.30E-004 | | 1.19E-002 | | 3.20E-004 | |
| IgG1AAG NtFX-KO (1) | | | | | 1.99E-007 | | 3.16E+006 | | | | 8.70E+005 | | | | | 0.8176 | | | | | 0.24 | | | | 0.008097 | | | 2.90E-004 | | 0.02672 | | 6.50E-004 | |
| IgG1AAG NtFX-KO (2) | | | | | 1.13E-007 | | 2.57E+006 | | | | 1.60E+005 | | | | | 0.2916 | | | | | 0.018 | | | | 1.13E-005 | | | 1.60E-005 | | 0.002333 | | 8.00E-004 | |
| IgG1AAG WT (1) | | | | | 7.76E-008 | | 8.06E+005 | | | | 1.70E+004 | | | | | 0.1169 | | | | | 0.003 | | | | 0.01112 | | | 3.60E-004 | | 0.0128 | | 3.80E-004 | |
| IgG1AAG WT (2) | | | | | 9.17E-008 | | 7.85E+005 | | | | 1.90E+004 | | | | | 0.1436 | | | | | 0.0047 | | | | 0.01371 | | | 4.20E-004 | | 0.01379 | | 3.40E-004 | |
| **Table S2 (continued)**  **A** | | |  | | |  | | | |  | | | | |  | | | | |  | | | |  | | |  | |  | |  | |  |
| **Antibody/ICI** | | | | | **Rmax (RU)** | | | **SE(Rmax)** | | | | **tc** | | | | | **SE(tc)** | | | | | **chi2** | | | |  |  |  |  |  |  |  |  |
| IgG1k MC | | | | | 62.8 | | | 8.2 | | | | 2.94E+007 | | | | | 9.30E+006 | | | | | 2.57 | | | |  |  |  |  |  |  |  |  |
| IgG1k NtFX-KO | | | | | 195 | | | 3.8 | | | | 3.24E+013 | | | | | 3.60E+017 | | | | | 4.84 | | | |  |  |  |  |  |  |  |  |
| IgG1AAG NtFX-KO (1) | | | | | 78.7 | | | 2.8 | | | | 2.84E+007 | | | | | 3.00E+006 | | | | | 0.139 | | | |  |  |  |  |  |  |  |  |
| IgG1AAG NtFX-KO (2) | | | | | 42.1 | | | 1.1 | | | | 1.63E+007 | | | | | 9.80E+005 | | | | | 0.997 | | | |  |  |  |  |  |  |  |  |
| IgG1AAG WT (1) | | | | | 229.1 | | | 2.7 | | | | 1.64E+015 | | | | | 1.10E+018 | | | | | 5.19 | | | |  |  |  |  |  |  |  |  |
| IgG1AAG WT (2) | | | | | 274.9 | | | 3.2 | | | | 3.50E+008 | | | | | 4.00E+007 | | | | | 6.57 | | | |  |  |  |  |  |  |  |  |
|  |  | **hFcRn pH 6.0 (2-state reaction model)** | | | | | | | | | | | |  | | | |  |  | | | |  |  |  |  |  |  |  |  |  |  |  |
| **Antibody/ICI** | | | | **K_D_** | | | | | **Rmax (RU)** | | | | **chi2** | | | | |  |  |  |  |  |  |  |  |  |  |  |  |  |  |  |  |
| IgG1k MC | | | | 3.101E-8 | | | | | 22.6 | | | | 0.431 | | | | |  |  |  |  |  |  |  |  |  |  |  |  |  |  |  |  |
| IgG1k NtFX-KO | | | | 2.173E-8 | | | | | 37 | | | | 1.73 | | | | |  |  |  |  |  |  |  |  |  |  |  |  |  |  |  |  |
| IgG4k MC (1) | | | | 1.085E-7 | | | | | 26.8 | | | | 0.197 | | | | |  |  |  |  |  |  |  |  |  |  |  |  |  |  |  |  |
| IgG4k MC (2) | | | | 7.168E-8 | | | | | 17 | | | | 0.328 | | | | |  |  |  |  |  |  |  |  |  |  |  |  |  |  |  |  |
| IgG4P MC (1) | | | | 8.931E-8 | | | | | 31.7 | | | | 0.288 | | | | |  |  |  |  |  |  |  |  |  |  |  |  |  |  |  |  |
| IgG4P MC (2) | | | | 1.619E-8 | | | | | 22.8 | | | | 0.976 | | | | |  |  |  |  |  |  |  |  |  |  |  |  |  |  |  |  |
| IgG4P NtFX-KO (1) | | | | 1.305E-8 | | | | | 20.6 | | | | 1.31 | | | | |  |  |  |  |  |  |  |  |  |  |  |  |  |  |  |  |
| IgG4P NtFX-KO (2) | | | | 1.069E-7 | | | | | 26 | | | | 2.51 | | | | |  |  |  |  |  |  |  |  |  |  |  |  |  |  |  |  |
| IgG4P WT (1) | | | | 1.143E-7 | | | | | 30.15 | | | | 0.213 | | | | |  |  |  |  |  |  |  |  |  |  |  |  |  |  |  |  |
| IgG4P WT (2) | | | | 1.117E-8 | | | | | 23.4 | | | | 1.45 | | | | |  |  |  |  |  |  |  |  |  |  |  |  |  |  |  |  |

**Table S2 (continued)**

**B**

**hFcRn pH 7.4 (2-state reaction model)**

| **Antibody/ICI** | **K_D_** |
| --- | --- |
| IgG1k MC | No binding |
| IgG1k NtFX-KO | No binding |
| IgG1AAG NtFX-KO (1) | No binding |
| IgG1AAG NtFX-KO (2) | No binding |
| IgG1AAG WT (1) | No binding |
| IgG1AAG WT (2) | No binding |

| **Antibody/ICI** | **K_D_** |
| --- | --- |
| IgG4k MC (1) | No binding |
| IgG4k MC (2) | No binding |
| IgG4P NtFX-KO (1) | No binding |
| IgG4P NtFX-KO (2) | No binding |
| IgG4P WT (1) | No binding |
| IgG4P WT (2) | No binding |

**Table S2 (continued)**

**C**

**hFcγRI**

1:1 langmuir model

| **Antibody/ICI** | **K_D_** | **Rmax (RU)** | **ka (1/Ms)** | **kd (1/s)** | **tc** | **chi2** | **U-value** |
| --- | --- | --- | --- | --- | --- | --- | --- |
| IgG1k MC (1) | 1.22E-009 | 69.4 | 1.47E+005 | 1.79E-004 | 2.64E+015 | 1.39 | 7 |
| IgG1k MC (2) | 7.27E-011 | 87.7 | 3.14E+006 | 2.28E-004 | 1.24E+008 | 2.19 | 2 |
| IgG1k NtFX-KO (1) | 1.41E-010 | 50.63 | 2.10E+006 | 2.95E-004 | 7.05E+014 | 1.38 | 2 |
| IgG1k NtFX-KO (2) | 1.63E-010 | 46.1 | 1.403E+006 | 2.280E-4 | 7.010E+21 | 0.228 | 1 |
| IgG1AAG MC (1) | 5.44E-009 | 3.615 | 4.29E+005 | 0.002333 | 1.51E+007 | 0.121 | 9 |
| IgG1AAG MC (2) | 1.94E-010 | 2.43 | 1.61E+006 | 3.13E-004 | 1.49E+018 | 0.316 | 26 |
| IgG1AAG NtFX-KO (1) | 6.21E-009 | 6.39 | 4.55E+005 | 0.002822 | 7.27E+007 | 0.219 | 4 |
| IgG1AAG NtFX-KO (2) | 4.75E-009 | 7.604 | 3.05E+006 | 1.45E-002 | 8.96E+005 | 0.247 | 26 |

2-state reaction model

| **Antibody/ICI** | **K_D_** | **ka1 (1/Ms)** | **SE(ka1)** | **kd1 (1/s)** | **SE(kd1)** | **ka2 (1/s)** | **SE(ka2)** | **kd2 (1/s)** | **SE(kd2)** |
| --- | --- | --- | --- | --- | --- | --- | --- | --- | --- |
| IgG1AAG WT (1) | 2.70E-007 | 4.83E+004 | 4.60E+003 | 0.05111 | 0.0018 | 0.004799 | 9.90E-005 | 0.001645 | 3.20E-005 |
| IgG1AAG WT (2) | 5.53E-009 | 8.37E+005 | 7.60E+004 | 0.0146 | 0.0018 | 0.002759 | 2.50E-004 | 1.28E-003 | 1.80E-004 |

| **Antibody/ICI** | **Rmax (RU)** | **SE(Rmax)** | **tc** | **SE(tc)** | **chi2** |
| --- | --- | --- | --- | --- | --- |
| IgG1AAG WT (1) | 164.5 | 15 | 5.10E+011 | 7.80E+010 | 0.139 |
| IgG1AAG WT (2) | 9.4 | 0.32 | 4.74E+008 | 1.20E+010 | 0.901 |

**Table S2 (continued)**

**D**

**hFcγRIIa (Steady state)**

| **Antibody/ICI** | **K_D_** | **Rmax (RU)** | **chi2** |  |  |  |  |  |
| --- | --- | --- | --- | --- | --- | --- | --- | --- |
| IgG1k MC (1) | 1.97E-007 | 84.86 | 0.466 |  |  |  |  |  |
| IgG1k MC (2) | 4.21E-007 | 17.26 | 0.143 |  |  |  |  |  |
| IgG1k NtFX-KO (1) | 6.29E-007 | 12.2 | 0.126 |  |  |  |  |  |
| IgG1k NtFX-KO (2) | 1.23E-008 | 100.3 | 18 |  |  |  |  |  |
| IgG1AAG MC (1) | No binding |  |  |  |  |  |  |  |
| IgG1AAG MC (2) | No binding |  |  |  |  |  |  |  |
| IgG1AAG NtFX-KO (1) | No binding |  |  |  |  |  |  |  |
| IgG1AAG NtFX-KO (2) | No binding |  |  |  |  |  |  |  |
| IgG1AAG WT (1) | No binding |  |  |  |  |  |  |  |
| IgG1AAG WT (2) | No binding |  |  |  |  |  |  |  |

**Table S2 (continued)**

**E**

**hFcγRIIb/c (2-state reaction model)**

| **Antibody/ICI** | **K_D_** | **Rmax (RU)** | **chi2** | |
| --- | --- | --- | --- | --- |
| IgG1k MC (1) | 3.76E-006 | 60.88 | 0.0302 |  |
| IgG1k MC (2) | 2.955E-006 | 216.1 | 3.53 |  |
| IgG1k NtFX-KO (1) | 6.006E-006 | 374.2 | 0.0521 |  |
| IgG1k NtFX-KO (2) | 1.72E-006 | 61.92 | 0.0334 |  |
| IgG1AAG MC (1) | No binding |  |  |  |
| IgG1AAG MC (2) | No binding |  |  |  |
| IgG1AAG NtFX-KO (1) | No binding |  |  |  |
| IgG1AAG NtFX-KO (2) | No binding |  |  |  |
| IgG1AAG WT (1) | 2.25E-005 | n.a. | 3.84 |  |
| IgG1AAG WT (2) | 9.03E-006 | 269.5 | 2.79 |  |

**Table S2 (continued)**

**F**

**hFcγRIIIa (2-state reaction model)**

| **Antibody/ICI** | **K_D_** | **ka1 (1/Ms)** | | **SE(ka1)** | | **kd1 (1/s)** | | **SE(kd1)** | | | **ka2 (1/s)** | **SE(ka2)** | **kd2 (1/s)** | **SE(kd2)** |
| --- | --- | --- | --- | --- | --- | --- | --- | --- | --- | --- | --- | --- | --- | --- |
| IgG1k MC (1) | 5.90E-007 | 2.60E+005 | | 9.70E+003 | | 0.1913 | | 8.40E-003 | | | 0.009031 | 4.90E-004 | 0.03622 | 9.60E-004 |
| IgG1k MC (2) | 2.44E-007 | 7.37E+005 | | 9.30E+004 | | 0.1888 | | 2.90E-002 | | | 1.84E-004 | 3.10E-005 | 0.003529 | 7.10E-004 |
| IgG1k NtFX-KO (1) | 2.12E-008 | 6.97E+005 | | 5.60E+003 | | 0.02007 | | 2.80E-004 | | | 0.003937 | 1.30E-004 | 0.01107 | 1.30E-004 |
| IgG1k NtFX-KO (2) | 3.98E-008 | 4.14E+005 | | 3.10E+003 | | 0.02323 | | 2.90E-004 | | | 0.004554 | 9.90E-005 | 0.01111 | 9.20E-005 |
| IgG1AAG MC (1) | 3.84E-006 | 2.24E+002 | | 6.60E+000 | | 0.00732 | | 3.10E-004 | | | 6.20E-003 | 2.10E-004 | 8.27E-004 | 3.70E-005 |
| IgG1AAG MC (2) | 6.66E-006 | 2.58E+002 | | 1.00E+001 | | 0.01043 | | 6.30E-004 | | | 0.00826 | 3.50E-004 | 0.001631 | 5.10E-005 |
| IgG1AAG NtFX-KO (1) | 1.91E-008 | 6.80E+005 | | 1.30E+006 | | 0.01331 | | 7.60E-002 | | | 2.76E-004 | 7.90E-003 | 0.01037 | 1.50E-002 |
| IgG1AAG NtFX-KO (2) | 1.29E-006 | 3.51E+002 | | 3.90E+002 | | 0.02998 | | 0.026 | | | 0.01249 | 5.60E-003 | 1.92E-004 | 8.20E-004 |
| IgG1AAG WT (1) | 2.38E-005 | 100.2 | | 6.5 | | 0.01745 | | 5.80E-004 | | | 0.008288 | 1.30E-004 | 0.001309 | 1.80E-005 |
| IgG1AAG WT (2) | 7.13E-007 | 3390 | | 1.50E+002 | | 0.01729 | | 4.50E-004 | | | 0.006428 | 8.70E-005 | 0.001045 | 1.80E-005 |
|  |  |  | |  | |  | |  | | |  |  |  |  |
| **Antibody/ICI** | **Rmax (RU)** | | **SE(Rmax)** | | **tc** | | **SE(tc)** | | **chi2** |  |  |  |  |  |
| IgG1k MC (1) | 47.6 | | 1.1 | | 2.91E+007 | | 9.80E+006 | | 0.11 |  |  |  |  |  |
| IgG1k MC (2) | 24 | | 1.2 | | 7.76E+006 | | 2.10E+006 | | 0.256 |  |  |  |  |  |
| IgG1k NtFX-KO (1) | 56.3 | | 0.11 | | 1.72E+008 | | 2.70E+007 | | 0.237 |  |  |  |  |  |
| IgG1k NtFX-KO (2) | 57.8 | | 0.12 | | 7.89E+007 | | 8.40E+006 | | 0.103 |  |  |  |  |  |
| IgG1AAG MC (1) | 567.4 | | 14 | | 9.79E+007 | | 2.10E+007 | | 0.0436 |  |  |  |  |  |
| IgG1AAG MC (2) | 529.8 | | 16 | | 9.41E+007 | | 1.30E+007 | | 0.0892 |  |  |  |  |  |
| IgG1AAG NtFX-KO (1) | 0 | | 0.0069 | | 1.00E+008 | | 6.20E+008 | | 0.088 |  |  |  |  |  |
| IgG1AAG NtFX-KO (2) | 3.2 | | 3.3 | | 1.00E+008 | | 1.10E+008 | | 0.147 |  |  |  |  |  |
| IgG1AAG WT (1) | n.a. | | 2.10E+002 | | 1.47E+008 | | 4.30E+007 | | 0.0875 |  |  |  |  |  |
| IgG1AAG WT (2) | n.a. | | 4.1 | | 2.66E+011 | | 1.40E+013 | | 0.0721 |  |  |  |  |  |

**Table S3. Probability (p) values of Fcγ receptor binding differences of antibodies and ICIs.** P-values indicating whether there were significant differences between binding of commercial IgG1k and IgG4k (Sigma, USA), IgG4P ICIs made in mammalian cells and wild-type (WT) or glycomodified (NtFX-KO) plants, as well as IgG1AAG ICIs made in WT or NtFX-KO plants to A) FcγRI, B) FcγRIIa, C) FcγRIIb/c and D) FcγRIIIa. Blank-subtracted binding of antibodies to Fcγ receptor (expressed as Response Units or RUs) were calculated as a percentage of IgG1k binding to the same receptor (IgG1k set as 1.0) and Mean ± SD were reported in Table 3B. Results in bold indicate p-values of p≤0.05. Results in asterisks (*) indicate p-values of 0.09>p>0.05. Three biological replicates were performed for each experiment.

**A**

**hFcγRI**

| **ICIs** | IgG1 | IgG4 | IgG4P (MC) | IgG4P (NtFX-KO) | IgG4P (WT) | IgG1AAG (NtFX-KO) | IgG1 AAG (WT) |
| --- | --- | --- | --- | --- | --- | --- | --- |
| IgG1 | - | 0.7977 | 0.6909 | 0.2935 | 0.5103 | **0.0049** | **0.0049** |
| IgG4 | - | - | 0.5419 | 0.2369 | 0.3948 | **0.0044** | **0.0044** |
| IgG4P (MC) | - | - | - | 0.4561 | 0.7931 | **0.0092** | **0.0092** |
| IgG4P (NtFX-KO) | - | - | - | - | 0.5978 | **0.0212** | **0.0211** |
| IgG4P (WT) | - | - | - | - | - | **0.0110** | **0.0110** |
| IgG1AAG (NtFX-KO) | - | - | - | - | - | - | 0.3868 |
| IgG1 AAG (WT) | - | - | - | - | - | - | - |

**Table S3 (continued)**

**B**

**hFcγRIIa**

| **ICIs** | IgG1 | IgG4 | IgG4P (MC) | IgG4P (NtFX-KO) | IgG4P (WT) | IgG1AAG (NtFX-KO) | IgG1 AAG (WT) |
| --- | --- | --- | --- | --- | --- | --- | --- |
| IgG1 | - | **0.0013** | **0.0431** | **0.0128** | **0.0045** | **0.0049** | **0.0048** |
| IgG4 | - | - | 0.1125 | **0.0189** | 0.3450 | 0.1075 | 0.1096 |
| IgG4P (MC) | - | - | - | **0.7016** | *0.0836 | *0.0553 | *0.0558 |
| IgG4P (NtFX-KO) | - | - | - | - | **0.0019** | **0.0037** | **0.0036** |
| IgG4P (WT) | - | - | - | - | - | **0.0187** | **0.0175** |
| IgG1AAG (NtFX-KO) | - | - | - | - | - | - | 0.4226 |
| IgG1 AAG (WT) | - | - | - | - | - | - | - |

**Table S3 (continued)**

**C**

**hFcγRIIb/c**

| **ICIs** | IgG1 | IgG4 | IgG4P (MC) | IgG4P (NtFX-KO) | IgG4P (WT) | IgG1AAG (NtFX-KO) | IgG1 AAG (WT) |
| --- | --- | --- | --- | --- | --- | --- | --- |
| IgG1 | - | 0.1064 | *0.0754 | *0.0737 | **0.0157** | **0.0063** | **0.0063** |
| IgG4 | - | - | 0.7318 | **0.8587** | *0.0585 | **0.0264** | **0.0264** |
| IgG4P (MC) | - | - | - | 0.6493 | *0.0565 | **0.0014** | **0.0014** |
| IgG4P (NtFX-KO) | - | - | - | - | *0.0683 | **<0.0001** | **<0.0001** |
| IgG4P (WT) | - | - | - | - | - | 0.3750 | 0.3750 |
| IgG1AAG (NtFX-KO) | - | - | - | - | - | - | >0.9999 |
| IgG1 AAG (WT) | - | - | - | - | - | - | - |

**Table S3 (continued)**

**D**

**hFcγRIIIa**

| **ICIs** | IgG1 | IgG4 | IgG4P (MC) | IgG4P (NtFX-KO) | IgG4P (WT) | IgG1AAG (NtFX-KO) | IgG1 AAG (WT) |
| --- | --- | --- | --- | --- | --- | --- | --- |
| IgG1 | - | **0.0021** | **0.0008** | **0.0066** | **0.0009** | **0.0007** | **0.0032** |
| IgG4 | - | - | 0.1191 | **0.0042** | 0.3786 | 0.2820 | 0.2114 |
| IgG4P (MC) | - | - | - | **0.0255** | 0.3692 | 0.5610 | *0.0795 |
| IgG4P (NtFX-KO) | - | - | - | - | **0.0215** | **0.0213** | **0.0243** |
| IgG4P (WT) | - | - | - | - | - | 0.7584 | 0.1656 |
| IgG1AAG (NtFX-KO) | - | - | - | - | - | - | 0.1461 |
| IgG1 AAG (WT) | - | - | - | - | - | - | - |

**Table S4. Input parameter settings used for the calculation of production costs of plant-derived ICIs.** Note (*) – the biomass per plant values were selected to span typical product recovery levels of *N. benthamiana* (~150 g) and *N. tabacum* (~350 g). Note (**) – a filter capacity of >1000 L m^-2^ that was previously reported (Buyel *et al.,* 2015) was not used because it relied on flocculants which have an unknown effect on the recovery of the ICI products investigated here. Note (***) – in addition to differences in environmental controls and thus accumulation differences, the calculation for the greenhouse scenario envisaged manual handling of plants, whereas a fully-automated setup was considered for the vertical farm scenario.

| **Parameter** | **Unit** | **Type** | **Value or Range** |
| --- | --- | --- | --- |
| Product quantity | [kg] | Numeric | 2–20 |
| Biomass per plant (*) | [kg] | Numeric | 0.15–0.35 |
| Plant age at harvest | [d] | Numeric | 40–56 |
| Product accumulation | [g kg^-1^] | Numeric | 0.01–2.00 |
| Filter capacity (**) | [L m^-2^] | Numeric | 50–300 |
| Recovery | [-] | Numeric | 0.05–1.00 |
| Safety factor | [-] | Numeric | 1.05 |
| Plants per batch | [-] | Numeric | 2000 |
| Expression strategy | [-] | Categoric | Transgenic; transient |
| Growth support | [-] | Categoric | Soil; stone wool |
| Growth environment (***) | [-] | Categoric | Greenhouse; vertical farm |
| Extraction method | [-] | Categoric | Blade-based; screw-press |

**Table S4 (continued)**

| **Parameter** | **Unit** | **Type** | **Value or Range** |
| --- | --- | --- | --- |
| Filter system | [-] | Categoric | 3-stage; 1-stage; integrated |
| Capture system | [-] | Categoric | Adsorber; packed-bed column |
| Polishing system | [-] | Categoric | Single-step; two-step |
| Labor cost | [€ h^-1^] | Numeric | 10–80 |
| Energy cost | [€ kWh^-1^] | Numeric | 0.15 |
| Fertilizer cost | [€ L^-1^] | Numeric | 0.02 |
| Fresh water cost | [€ L^-1^] | Numeric | 0.005 |
| Waste water cost | [€ L^-1^] | Numeric | 0.115 |
| Consumable cost | [€] | Numeric | diverse |
| Cost threshold CHO-derived ICIs | [€ g^-1^] | Numeric | 2500–6000 |

**
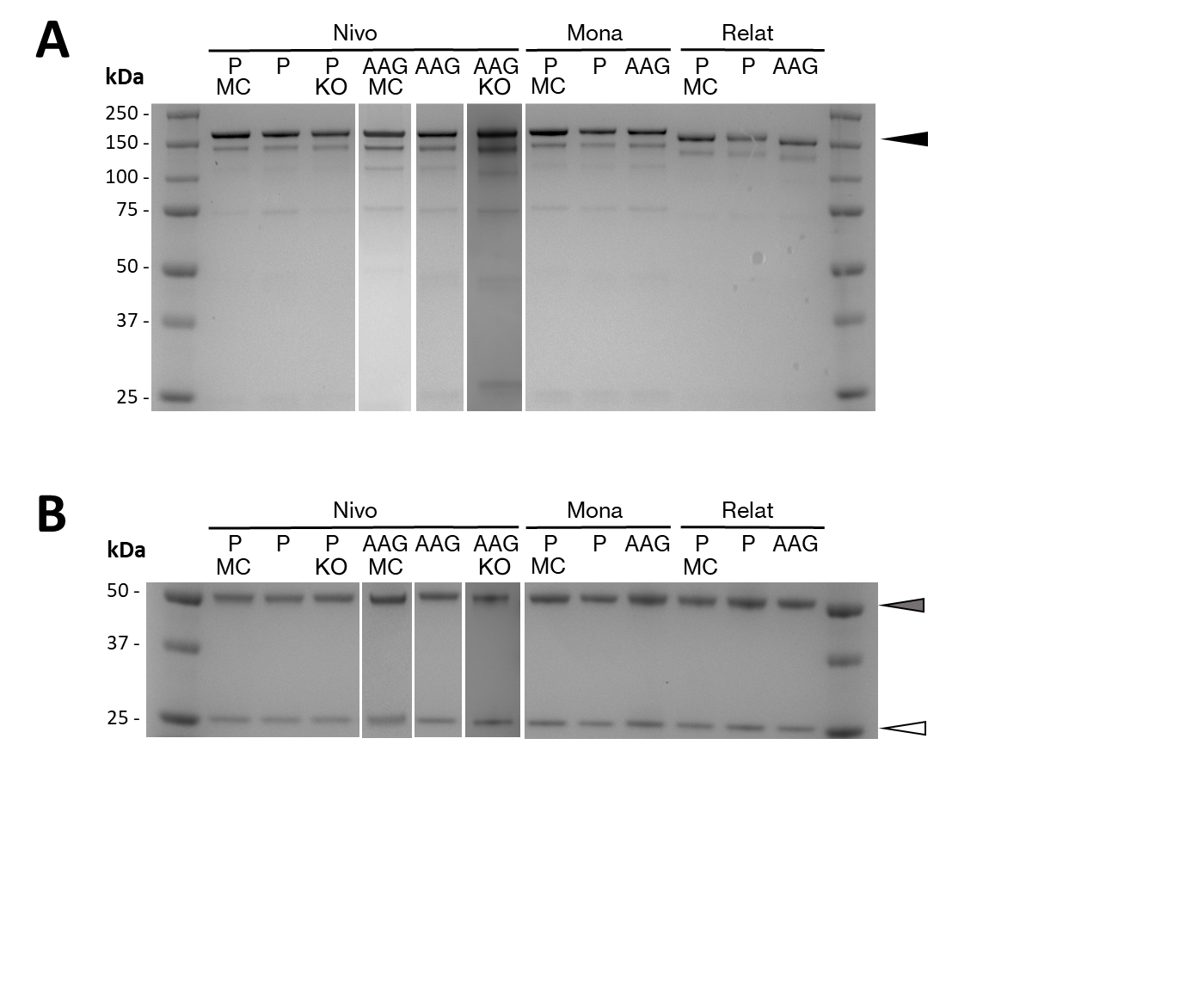
**

**Figure S1. PAGE gel of purified ICIs.** Purified ICIs Nivolumab (Nivo), Monalizumab (Mona) and Relatimab (Relat) with either IgG4 S228P (P) or IgG1 L324A/L325A/P329G (AAG) on a non-reducing (A) or reducing (B) PAGE gel. ICIs were produced in mammalian cells (MC), *N. benthamiana* with wild-type glycosylation, or afucosylated and axylosylated *N. tabacum* line (NtFX-KO; shortened to KO). Commercial mammalian-cell produced Nivolumab (Nivo MC; Absolute Antibody, USA) was used as positive control. Black arrow at 150 kDa indicate intact, correctly assembled ICIs. The grey arrow on the reducing gel indicate the heavy chain at 50 kDa, while the white arrow at 25 kDa indicate the light chain. 2 μg of purified ICIs were loaded onto the PAGE gels for non-reducing blots and 1 μg for reducing blots.

**
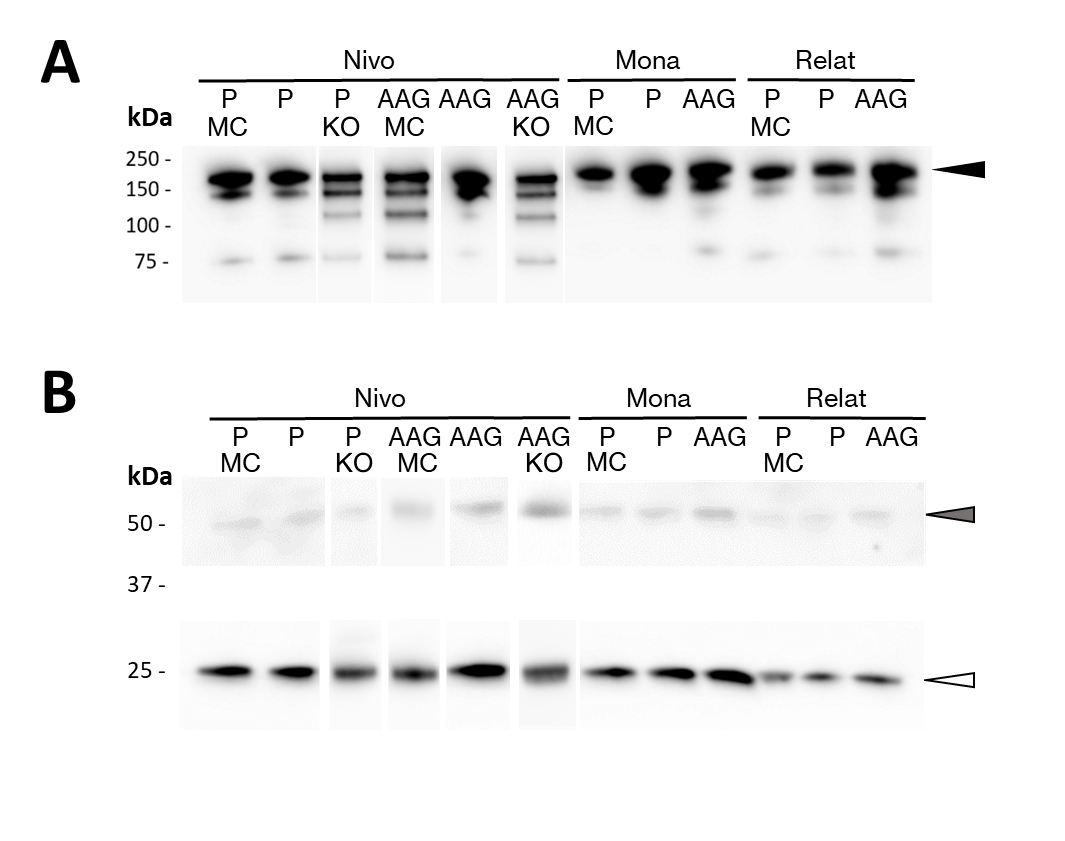
**

**Figure S2. Western blot of purified ICIs.** Purified ICIs Nivolumab (Nivo), Monalizumab (Mona) and Relatimab (Relat) with either IgG4 S228P (IgG4P) or IgG1 L324A/L325A/P329G (IgG1AAG) on a non-reducing (A) or reducing (B) Western blot. ICIs were produced in mammalian cells (MC), *N. benthamiana* with wild-type glycosylation, or afucosylated and axylosylated *N. tabacum* line (NtFX-KO; shortened to KO). Commercial mammalian-cell produced Nivolumab (Nivo MC; Absolute Antibody, USA) was used as positive control. Anti-human IgG gamma chain antiserum were used on the non-reducing and reducing Western blot to detect ICI heavy chain (black and grey arrows); while anti-kappa antisera was used to detect ICI light chain in reducing blot (white arrow). 50 ng of purified ICIs were loaded onto the blot.

**
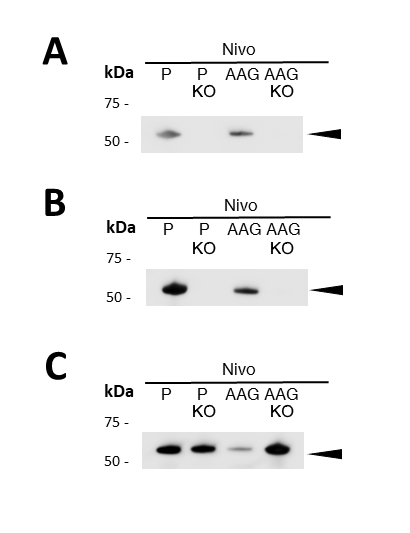
**

**Figure S3. Glycosylation of ICIs.** Nivolumab variants were presented as representatives of all ICIs. Western blot of IgG4 S228P (IgG4P) or IgG1 L324A/L325A/P329G (IgG1AAG) variants of Nivolumab produced in *N. benthamiana* with wild-type glycosylation or afucosylated and axylosylated *N. tabacum* line (NtFX-KO; shortened to KO). Anti-β1,2-xylose (A), anti-α1,3-fucose (B) and anti-human gamma (C) anti-sera were used to detect glycosylation on the heavy chain or the heavy chain itself. 50-100 ng of purified ICIs were loaded onto the blot.

**
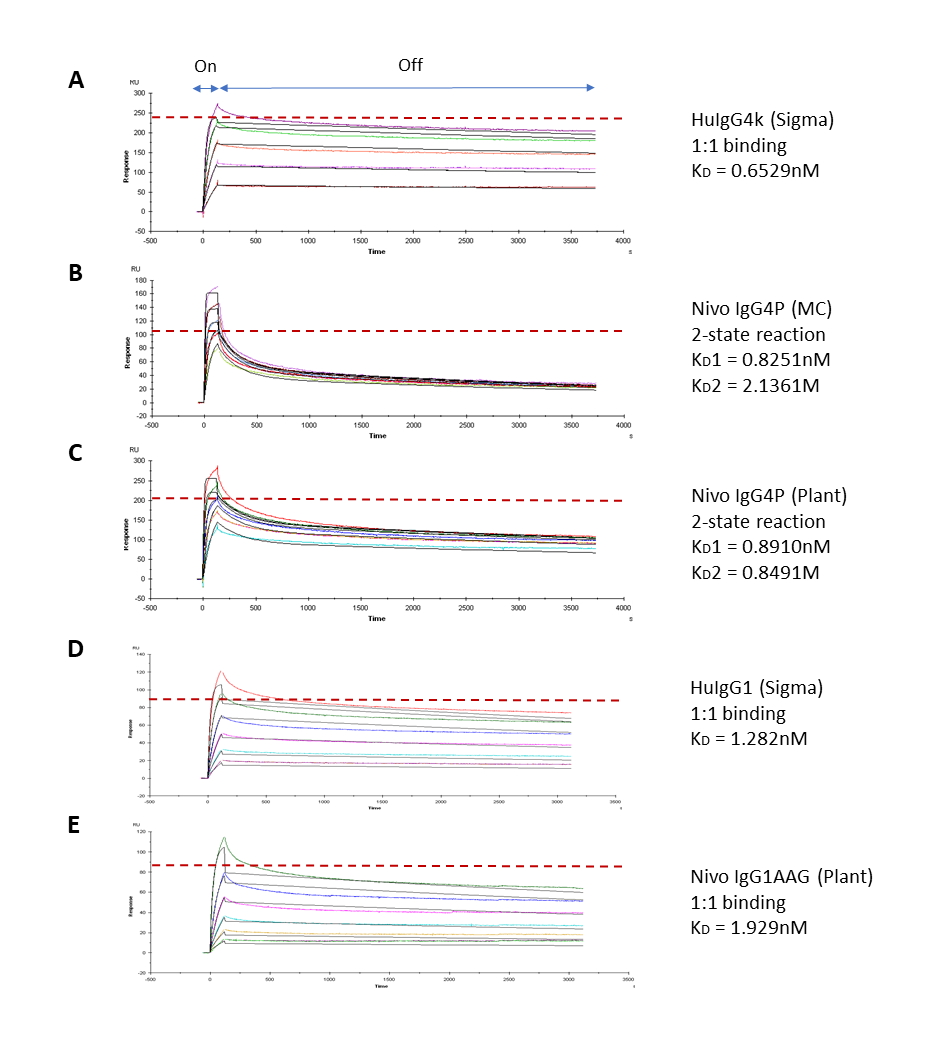
**

**Figure S4.** **Binding of ICIs to *S. aureus* Protein A.** Representative SPR sensorgrams showing the binding kinetics of *S. aureus* Protein A (Sigma, USA) to A) IgG4k from human myeloma plasma (Sigma, USA); B) mammalian-cell made Nivolumab IgG4P (Absolute Antibody, USA); C) plant-made Nivolumab IgG4P with wild-type glycosylation; D) IgG1k from human myeloma plasma (Sigma, USA); and E) plant-made Nivolumab IgG1AAG with wild-type glycosylation. Red dashed lines indicate R_max_. On: Association; Off: Dissociation

**
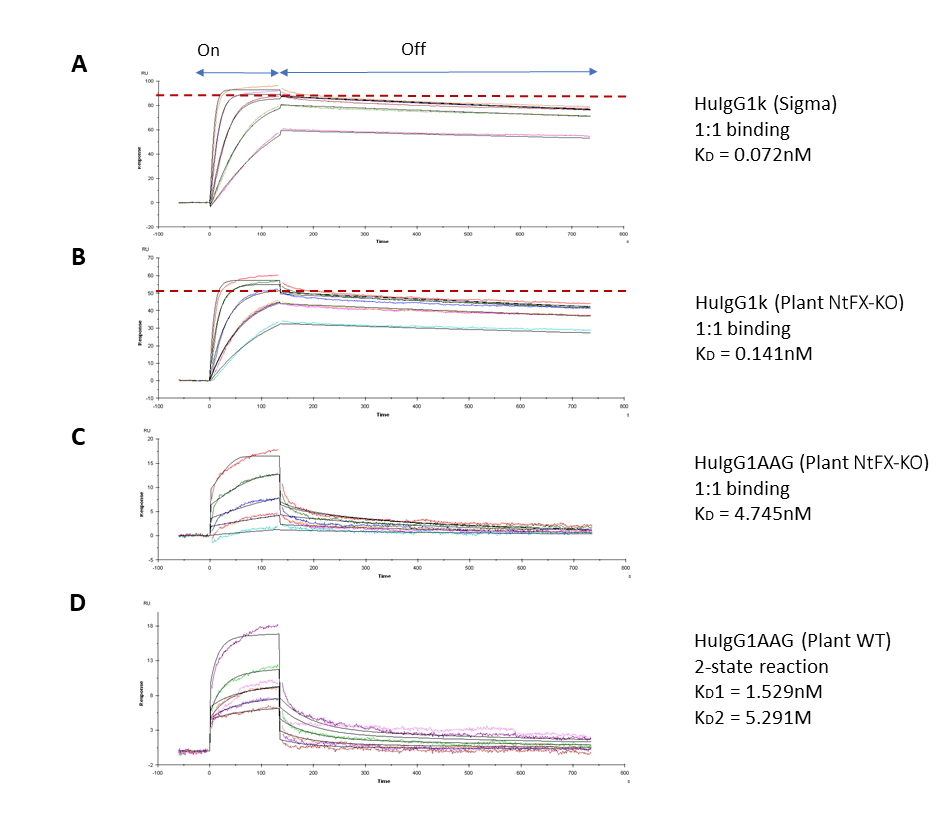
**

**Figure S5. Binding of antibodies and ICIs to human FcγRI receptor.** Representative SPR sensorgrams showing the binding kinetics of human FcγRI receptor (R&D Systems, USA) to A) commercial human IgGk1 from myeloma cells (Sigma, USA); B) non-related human IgG1k made in glycomodified *N. tabacum* plants (NtFX-KO); C) IgG1AAG ICI made in NtFX-KO plants; and D) IgG1AAG ICI made in plants with wild-type (WT) glycosylation. Red dashed lines if present indicate R_max_ of >50 RU has been reached. On: Association; Off: Dissociation.

**
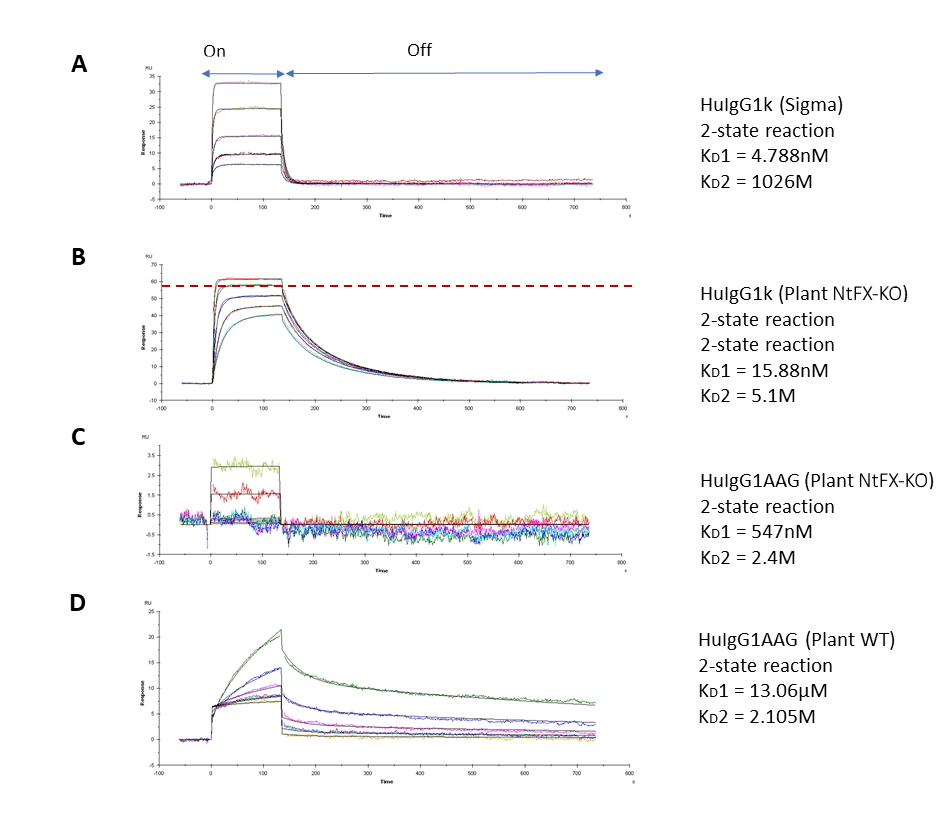
**

**Figure S6. Binding of antibodies and ICIs to human FcγRIIIa receptor.** Representative SPR sensorgrams showing the binding kinetics of human FcγRIIIa receptor (R&D Systems, USA) to A) commercial human IgGk1 from myeloma cells (Sigma, USA); B) non-related human IgG1k made in glycomodified *N. tabacum* plants (NtFX-KO); C) IgG1AAG ICI made in NtFX-KO plants; and D) IgG1AAG ICI made in plants with wild-type (WT) glycosylation. Red dashed lines if present indicate R_max_ of >50 RU has been reached. On: Association; Off: Dissociation.

**
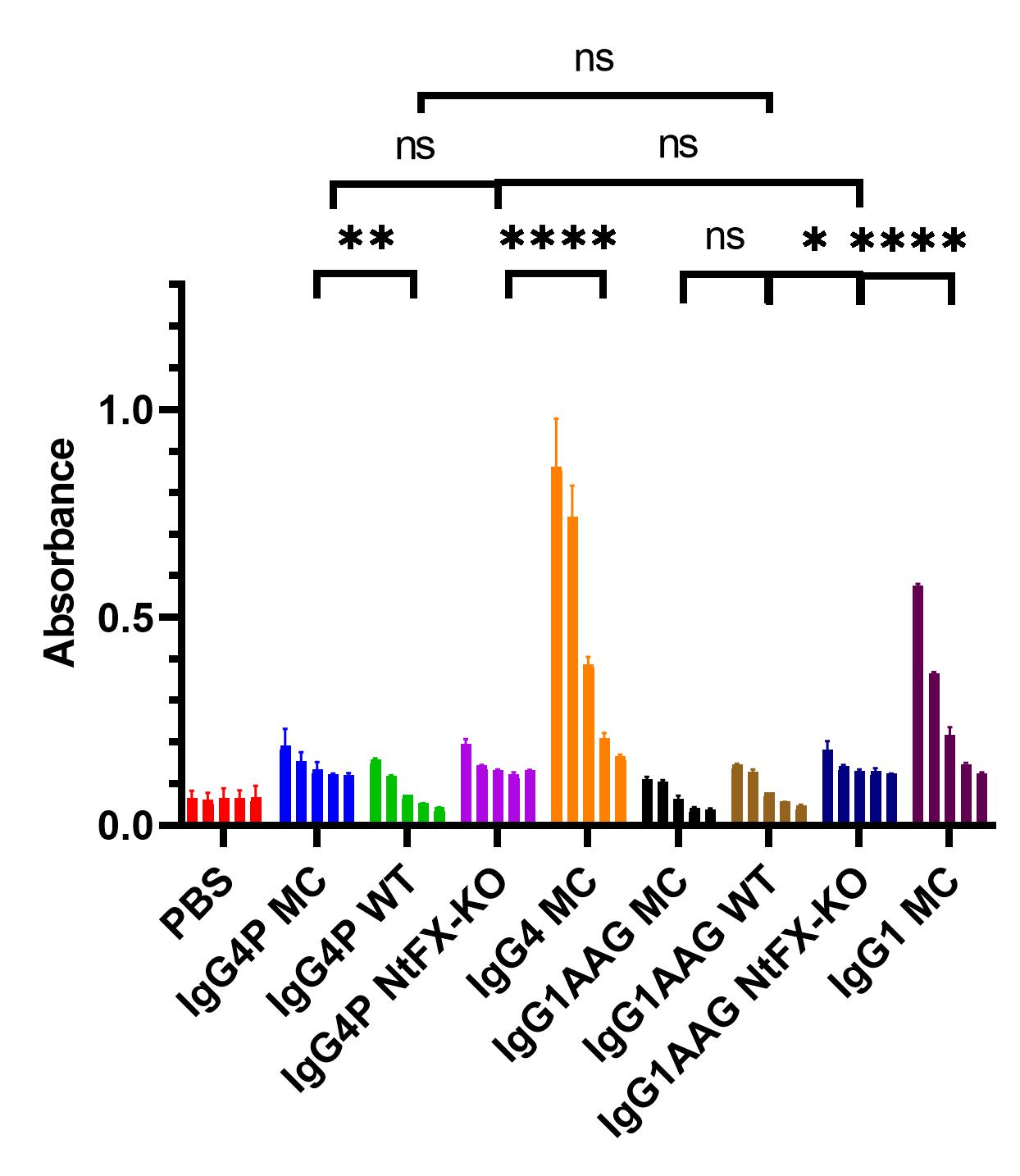
**

**Figure S7. Binding of ICIs to hC1q.** Binding of IgG4P- and IgG1AAG ICIs made in *N. benthamiana* plants with wild-type glycosylation (WT), or afucosylated and axylosylated *N. tabacum* (NtFX-KO), to human complement component C1q. PBS was used a negative controls and non-Fc modified IgG4 or IgG1 and Fc-modified IgG4P and IgG1AAG from mammalian cells were used as positive controls. Graph showing mean and standard deviation of three biological repeats.

**
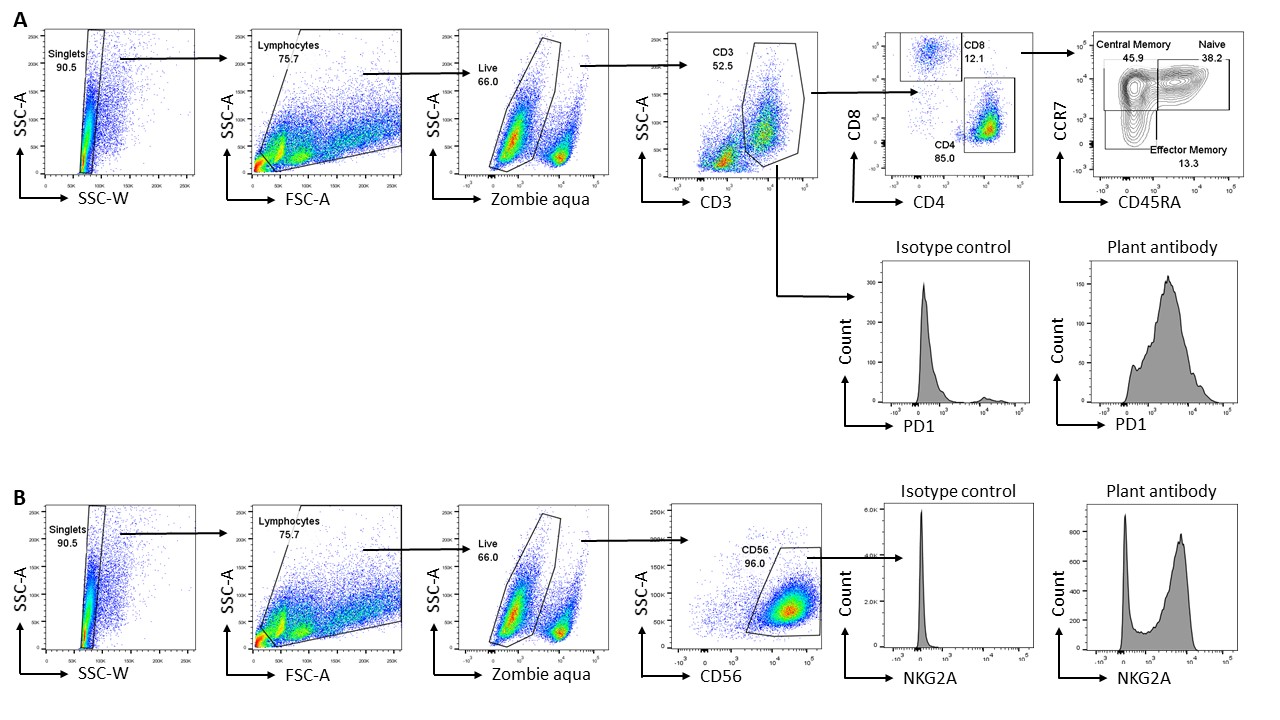
**

**Figure S8. Gating strategy for PD-1, LAG-3, NKG2A expression.** Doublets were excluded by SSC-A versus SSC-W and lymphocytes were gated based on SSC-A versus FSC-A. From this, live cells were gated based on negativity for zombie aqua staining. A) In isolated and activated T-cells CD3 positive cells were gated and from this population PD-1 and LAG-3 expression was examined. PD-1 and LAG-3 positivity was determined by gates set based on expression within IgG isotype controls. From CD3 positive cells both CD4 positive and CD8 positive cells were identified and T-cell sub-populations were gated based on CD45RA versus CCR7. Naïve cells were CD45RA+CCR7+, central memory cells were CD45RA-CCR7+ and effector memory cells were CD45RA-CCR7-. PD-1 and LAG-3 positive gates were then copied onto the relevant T-cell sub-populations. B) In isolated and activated NK cells CD56 positive cells were gated from the live cell gate and from this population NKG2A expression was assessed as histograms

**
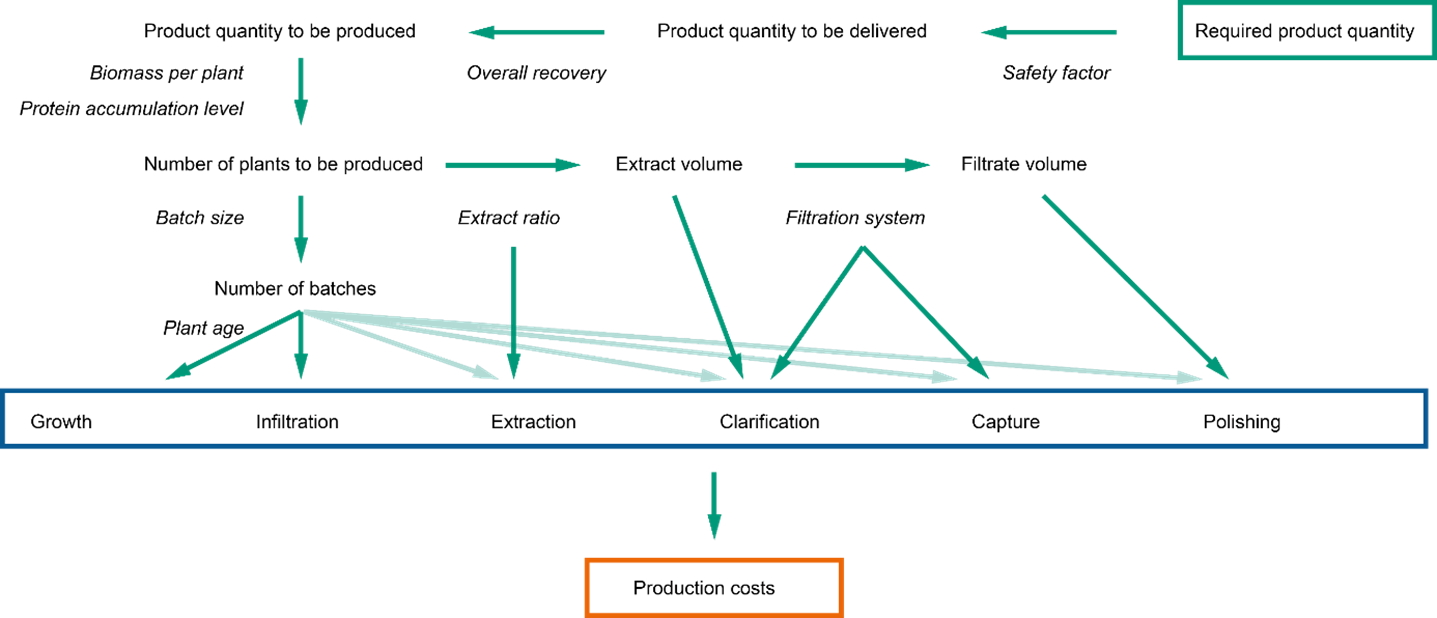
**

**Figure S9. Top-down model workflow to calculate the production costs per gram of ICI.** Starting from the product quantity to be delivered, losses were accounted for by a safety factor and process recovery resulting in the quantity of ICI to be produced. This value was converted into upstream production and downstream purification costs based on number of plants to be cultivated and biomass to be processed respectively. The dominant parameter determining both costs was the product accumulation level in the biomass. Parameter values and ranges used in the model are listed in Table S4.

**References (Supplementary materials)**

Buyel, J.F., Opdensteinen, P., and Fischer, R. (2015) Cellulose-based filter aids increase the capacity of depth filters during the
 downstream processing of plant-derived biopharmaceutical proteins. Biotechnol. J., **10**, 584–591.
